# A computer-guided design tool to increase the efficiency of cellular conversions

**DOI:** 10.1038/s41467-021-21801-4

**Published:** 2021-03-12

**Authors:** Sascha Jung, Evan Appleton, Muhammad Ali, George M. Church, Antonio del Sol

**Affiliations:** 1 Computational Biology Group, CIC bioGUNE-BRTA (Basque Research and Technology Alliance), Bizkaia Technology Park, Derio, Spain; 2 Wyss Institute for Biologically Inspired Engineering at Harvard University, Boston, MA USA; 3 Department of Genetics, Harvard Medical School, Boston, MA USA; 4 Computational Biology Group, Luxembourg Centre for Systems Biomedicine (LCSB), University of Luxembourg, Esch-sur-Alzette, Luxembourg; 5 Maastricht University School for Mental Health and Neuroscience (MHeNs), Department of Psychiatry and Neuropsychology, Maastricht University, Maastricht, the Netherlands; 6 GC Therapeutics, Inc, Cambridge, MA USA; 7 IKERBASQUE, Basque Foundation for Science, Bilbao, Spain; 8 Moscow Institute of Physics and Technology, Dolgoprudny, Russia

**Keywords:** Computational models, Data integration, Gene regulatory networks, Reprogramming, Logic gates

## Abstract

Human cell conversion technology has become an important tool for devising new cell transplantation therapies, generating disease models and testing gene therapies. However, while transcription factor over-expression-based methods have shown great promise in generating cell types in vitro, they often endure low conversion efficiency. In this context, great effort has been devoted to increasing the efficiency of current protocols and the development of computational approaches can be of great help in this endeavor. Here we introduce a computer-guided design tool that combines a computational framework for prioritizing more efficient combinations of instructive factors (IFs) of cellular conversions, called IRENE, with a transposon-based genomic integration system for efficient delivery. Particularly, IRENE relies on a stochastic gene regulatory network model that systematically prioritizes more efficient IFs by maximizing the agreement of the transcriptional and epigenetic landscapes between the converted and target cells. Our predictions substantially increased the efficiency of two established iPSC-differentiation protocols (natural killer cells and melanocytes) and established the first protocol for iPSC-derived mammary epithelial cells with high efficiency.

## Abstract (web summary)

Transcription factor over-expression-based cellular conversion methods often endure low conversion efficiency. Here the authors show how to increase conversion efficiency by combining a computational method for prioritizing more efficient TF combinations with a transposon-based genomic integration system for delivery.

## Introduction

Cellular conversion technologies are the key to human disease modeling, cell transplantation, and gene therapies, all of which require the efficient generation of a wide range of different human cell types^1–3^. Since it is often difficult to identify, purify, and expand many primary human cell types that can be readily used in this context, scientists have been investigating methods for converting a cell type that can be easily obtained and expanded efficiently to another cell type. For instance, in the context of ex vivo gene therapies, gene-corrected patient-derived induced pluripotent stem cells (iPSCs) have been differentiated into a variety of cell types, such as keratinocytes and epidermal pigment cells, and successfully transplanted^4,5^. While some recent work has been done in this area that mainly uses a variety of media-based and transcription factor (TF) over-expression-based cell culture methods, there are still no robust general methods for optimizing selections of TFs for high conversion efficiency.

Although there is compelling evidence that only a small set of over-expressed TFs are sufficient to confer cell identity and are being used for cellular conversion, which we refer to as instructive factors (IFs), this process is usually inefficient^6^. In particular, conversion efficiency refers to the ratio of successfully converted cells to the initial number of cells, a widely adopted definition we preserve in the remainder of this manuscript. In this context, four major determinants of conversion efficiency have been highlighted in recent studies. First, in addition to identity TFs, certain co-factors have to be up-regulated that cooperatively induce the target cell type^7^. Second, cell conversion efficiency is crucially dependent on the amount of epigenetic restructuring of the initial cell type during the conversion process^8,9^. Third, the conversion efficiency is influenced by inherent stochastic activation of co-factors^10^, and fourth, in addition to the combination of IFs, current protocols mostly rely on viral vectors for factor delivery, which results in limited cargo capacity and diminished conversion efficiency^11^. These determinants are further supported by recent computational studies emphasizing the importance to consider the epigenetic landscape in cellular conversions^12–15^. For instance, a computational model of epigenetic regulation underscored the importance of stochasticity and epigenetic regulation demonstrating that differentiation of pluripotent cells can be induced by solely altering the kinetics of epigenetic regulators and, thus, the epigenetic landscape^12^.

The identification of IFs for cellular conversions has prompted the development of computational methods to guide experimental efforts. Early approaches relied on the identification of significant differences in transcriptomic or epigenetic profiles^16–19^ while more recent methodologies combined transcriptomic data with gene regulatory network (GRN) reconstruction^20,21^. However, none of these methods account for the major determinants of conversion efficiency and, thus, are unable to systematically predict IFs for inducing efficient cellular conversions. Indeed, based on experimental evidence, gene expression alone is presumably insufficient for determining efficient IFs^22–24^.

Here, we present a computer-guided design tool for increasing the percentage of successfully converted cells, which addresses all four major determinants of conversion efficiency. The computational part of this design relies on an Integrative gene REgulatory NEtwork model (IRENE) that systematically integrates gene expression, histone modification, chromatin accessibility, TF ChIP-seq, and protein–protein interaction (PPI) data to reconstruct cell-type-specific core GRNs composed of identity TFs and their co-factors. Based on these cell-type-specific core GRNs, IRENE employs a stochastic Markov Chain approach to computationally simulate cellular conversion and identify optimal combinations of IFs, whose over-expression at the initial cell type maximizes agreement at the transcriptional and epigenetic levels between the converted and target cells. Results showed that IRENE predicted a larger number of known IFs in 29 examples of human cellular conversions in comparison to other state-of-the-art methods and correctly discerned predictions of high and low-efficiency IFs in eight previously experimentally validated examples of cellular reprogramming. Furthermore, the experimental part of the design uses piggyBac-integrable^25^ TF-over-expression cassettes via the human TFome^26^ to upregulate the predicted IF combinations by IRENE without concern of genetic silencing. Using this computer-guided design tool we increased the efficiency of two established human iPSC-differentiation protocols for natural killer cells and melanocytes up to ninefold and established the first protocol for human iPSC-derived mammary epithelial cells with high efficiency. In summary, we demonstrate that this tool offers the most accurate and efficient method to date for using TFs in direct cell-type conversions and is expected to significantly enhance the production of cell sources for cell transplantations and gene therapies.

## Results

### Reconstruction of cell-type-specific core GRNs

We propose a computer-guided design tool for TF over-expression-based cellular conversions to overcome the abiding issue of conversion efficiency. For that, we developed IRENE, a computational framework that models the major determinants of conversion efficiency and prioritizes more efficient sets of IFs (Supplementary Fig. 1). IRENE identifies these IFs by integrating transcriptomic and epigenetic profiles along with publicly available TF binding sites and enhancer-promoter interactions to reconstruct cell-type-specific core GRNs. For each TF, active enhancer and promoter regions are established by combining enhancer-promoter interactions from GeneHancer^27^ with cell-type-specific H3K27ac peaks and identifying H3K4me3 peaks around transcription start sites (TSS), respectively. IRENE filters these regions by overlaying cell-type-specific DNase-seq peaks to determine regulatory binding events within these regions and reconstructs transcriptional regulators from over 224 million TF ChIP-seq peaks. Finally, IRENE identifies a set of 10 identity TFs by computing the TFs with the highest cell-type-specific expression in comparison to 7600 phenotypes using a modified version of Jensen-Shannon-Divergence (JSD)^16^. In addition, TFs fulfilling the following three conditions are included as co-factors of these identity TFs. First, each co-factor has to be significantly expressed. Second, it has to be regulated by at least one of the identified identity TFs and, third, it has to regulate at least one identity TF. Of note, IRENE does not impose a maximum number of co-factors. Thus, all TFs fulfilling these criteria are included in the network. Finally, the core GRN is composed of all regulatory interactions between identity TFs and their co-factors.

**Fig. 1:**
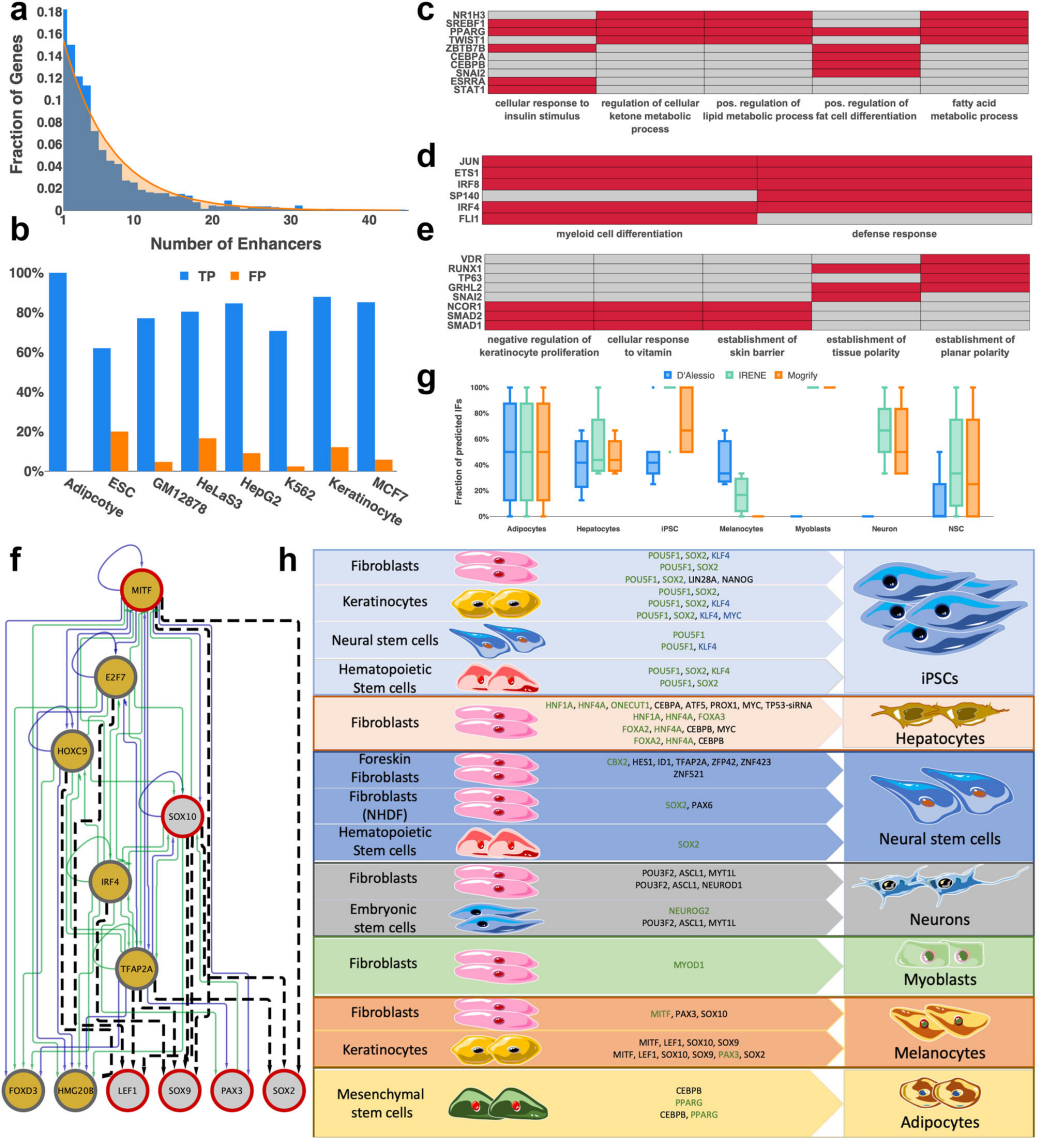
Benchmarking of IRENE. **a** The number of enhancers per gene (blue) across all networks follows an exponential distribution (orange). **b** Benchmark of reconstructed networks against cell-type-specific TF ChIP-Seq data for 8 cell types/cell lines. True positives (TP, blue) represent the interactions that are present in the reconstructed GRNs and are experimentally validated by cell-type-specific TF ChIP-Seq data. Interactions validated by TF ChIP-Seq data only profiled in cell-types other than the one under consideration are considered false positives (FP, orange). *n* = 13 (Adipocyte), 69 (ESC), 236 (GM12878), 238 (HeLaS3), 292 (HepG2), 53 (K562), 37 (Keratinocyte) and 178 (MCF7) interactions. **c**–**e** Most-highly enriched significant gene ontology terms for the reconstructed networks of **c** adipocytes, **d** natural killer cells and **e** mammary epithelial cells. For adipocytes and mammary epithelial cells, the top 5 GO terms are represented. For natural killer cells, only two terms were significantly enriched. Cells corresponding to TFs that are and are not relevant for a particular GO term are colored in red and gray, respectively. **f** Reconstructed melanocyte subnetwork including all experimentally validated (red border) and predicted IFs (gold) for the conversion of fibroblasts towards melanocytes. Enhancer and promoter regulation (green) is distinguished from enhancer-only regulation (blue). Predicted interactions from position weight matrices using Homer are depicted as black dashed lines. **g** Recovery of experimentally validated IFs in seven target cell types using IRENE (green), Mogrify (orange), and the method from d’Alessio et al. (blue). The fraction of recovered IFs in multiple combinations of cellular conversions is depicted as box plots. The median is represented by a solid line within the boxes. The lower and upper bounds of boxes are the first and third quartile, respectively. Whiskers extend to 1.5-times the interquartile range or the minimum/maximum value. Dots correspond to outliers. *n* = 3 (Adipocytes), 4 (Hepatocytes), 10 (iPSC), 3 (Melanocytes), 1 (Myoblasts), 4 (Neuron), and 4 (NSC) combinations of cellular conversion factors. **h** Enrichment of predicted instructive factors in experimentally validated IF combinations. Predicted IFs are highlighted in green whereas TFs that were replaced by another validated and more efficient IF are highlighted in blue. TFs not predicted by IRENE are colored in black.

We employed IRENE to reconstruct core GRNs for 72 human cell types, cell lines, and tissues. Every network has up to 51 TFs (on average 18.5 TFs), while every TF in the network has up to 46 regulators (on average 15.0) and 44 active enhancers (on average 6.0). The number of enhancers per gene follows an exponential distribution where the majority of genes have one or two active enhancers, which is consistent with enhancer-promoter interactions obtained from promoter capture Hi-C experiments^28^ (Fig. 1a). Moreover, unlike co-factors, core TFs are always differentially expressed between the initial and final cell types according to commonly used criteria (fold change > 2). Nevertheless, although co-factors are not necessarily differentially expressed, they are equally likely to be contained in the predicted IF combinations, since their over-expression could be beneficial to overcome the transcriptional and epigenetic barriers (Supplementary Table 1).

### Validation of reconstructed GRNs

Before employing IRENE’s reconstructed networks to generate predictions of IFs for efficient cellular conversions, we interrogated their accuracy and cell-type-specificity. For that, we first examined whether the set of selected identity TFs and co-factors is implicated in the functionality of the cell or tissue type. Significantly enriched gene ontology (GO) terms of the network TFs were identified using WebGestalt^29^ and showed a highly specific enrichment for most cell or tissue types (Supplementary Data 1). For instance, subcutaneous adipocytes were enriched in positive regulation of fat cell differentiation, natural killer cells were enriched in defense response while mammary epithelial cells were enriched in the establishment of the skin barrier (Fig. 1c–e).

Next, we validated the reconstructed interactions among network TFs. In the presence of incomplete ground truth data, we first assessed the number of interactions within promoter regions that are compatible with cell-type-specific TF ChIP-seq data from ChIP-Atlas^30^ (Supplementary Note 1). Requiring a representative evaluation of at least 10 network TFs resulted in eight examples of different cell types and cell lines. We evaluated a total of 1044 TF ChIP-seq experiments and validated on average 80.98% of interactions whereas 8.84% of interactions were “false positives”, i.e., regulatory binding events only occurring in a cell type other than the target (Fig. 1b). Afterwards, we collected four experimentally validated, manually curated gold-standard networks of embryonic stem cells (ESCs)^31^, hepatocytes^32^, HepG2, and MCF7 cells^33^ to compare them against reconstructed networks from IRENE. Of note, only TFs common to the reconstructed and gold-standard networks were considered in this assessment. In particular, 79% of TFs in the gold-standard networks are, on average, present in the reconstructed networks by IRENE (range: 50–100%) (Supplementary Table 2, Supplementary Data 2). Moreover, we observed that the networks for ESCs, HepG2, and MCF7 cells were in perfect agreement whereas a single interaction was missing in the reconstructed hepatocyte network (Table 1). Moreover, IRENE inferred four new interactions of HNF1A and FOXA2 in the hepatocyte network that have been validated in TF knockdown studies of hepatoma cells^34^. Thus, 95% of interactions in the gold-standard networks were correctly reconstructed, which highlights IRENE’s accuracy. In addition, we set out to validate the choice of databases underlying IRENE and performed the same assessment using enhancer-gene associations from EnhancerAtlas^35^ and transcriptional regulatory interactions from GTRD^36^. Indeed, using the data from EnhancerAtlas and GTRD, we could only validate 52% of interactions in the gold-standard networks, which supports the choice of databases underlying IRENE (Supplementary Table 2, Supplementary Data 2).

**Table 1:** Benchmarking reconstructed core GRNs against experimentally validated core networks.

Cell type	GS interactions	Inferred interactions	Matching interactions	Mismatching interactions	Newly inferred	Newly inferred (validated)	Overall validated
ESC	9	9	9	0	0	0	100%
Hepatocytes	13	12	8	2	4	4	85.71%
HepG2	16	16	16	0	0	0	100%
MCF7	13	4	4	0	0	0	100%

For four well-characterized human cell types and cell lines, the reconstructed core networks were compared against their experimentally validated gold-standard (GS) core networks. The column ‘newly inferred’ refers to the number of interactions not present in the gold-standard network whereas the column ‘Newly inferred (validated)’ refers to how many of them were validated in literature.

### Prediction of IFs for inducing cellular conversions

Considering the stochastic nature of cellular conversions, we set out to convert reconstructed GRNs by IRENE into Deterministic Time Markov Chain models (DTMCs) that we can exploit for interrogating the dynamics of the system. For that, Boolean expressions were defined that connect the regulators of a TF and represent their competitive or cooperative action. IRENE characterizes two regulatory events as cooperative if their corresponding ChIP-seq peaks significantly overlap and an experimentally validated protein-protein interaction was reported in iRefIndex^37^ (see “Methods”). Otherwise, regulatory events are deemed competitive. Using these models, we developed a strategy for identifying combinations of TFs that induce cellular conversions with increased efficiency. In brief, IRENE identifies combinations of TFs whose over-expression at the initial cell type maximizes the agreement at the transcriptional and epigenetic level between the converted and target cells. To achieve this, IRENE assesses the probability that a perturbation activates the complete network of the target cell type and considers the amount of epigenetic restructuring needed to transform the enhancer/promoter landscape of the initial to the target cell type (see “Methods”).

To begin with, we assessed whether IRENE’s strategy to prioritize combinations of TFs is able to recapitulate known IFs. Starting from a collection of 29 human cell conversion experiments for which epigenetic and transcriptomic profiles were available, we first assessed the number of recovered IFs (Fig. 1h). Next, we compared our predictions against two former state-of-the-art approaches, Mogrify^20^ and d’Alessio et al.^16^. Indeed, IRENE substantially outperforms Mogrify and d’Alessio et.al, exhibiting median accuracy of 83.3% compared to 50% and 33.3%, respectively (Fig. 1g). Moreover, we observed a remarkable enrichment of predicted TFs for iPSCs, showing on average 95% recovery of known IFs compared to 72.5 and 45% with Mogrify and d’Alessio et al. (Fig. 1g).

Despite the overall increased performance, IRENE’s predictions of melanocytes were vastly inconsistent (17%), which prompted us to investigate this case more closely. Only three of the known IFs are included in the reconstructed melanocyte GRN, namely MITF, SOX10, and PAX3 (Fig. 1f). However, binding site predictions of known motifs from Homer^38^ in the promoter regions of known IFs confirmed many network TFs as upstream regulators. Importantly, one of the predicted TFs, TFAP2A, displays predicted binding sites within the promoter region of multiple IFs (Fig. 1f). In the presence of a recent study showing that TFAP2A is likely a pioneer factor capable of establishing competence for transcription, it is highly probable that TFAP2A could more efficiently induce melanocyte conversion^39^.

### IRENE prioritizes more efficient combinations of IFs

Given that IRENE resembled a majority of known IFs and at the same time predicted other combinations, we investigated whether IRENE prioritizes combinations yielding higher cellular conversion efficiency. For that, we collected examples of IF combinations inducing the same transition with different efficiency. In order to assess the real contribution of the IFs on conversion efficiency, we required the combinations to be reported in the same study using the same experimental design as well as all IFs to be present in the reconstructed GRNs. As a result, only iPSC conversion fulfilled both of these criteria. In particular, we identified eight pairs of IFs fulfilling our inclusion criteria in which the efficiency was assessed and focused on these transitions.

**Fig. 2:**
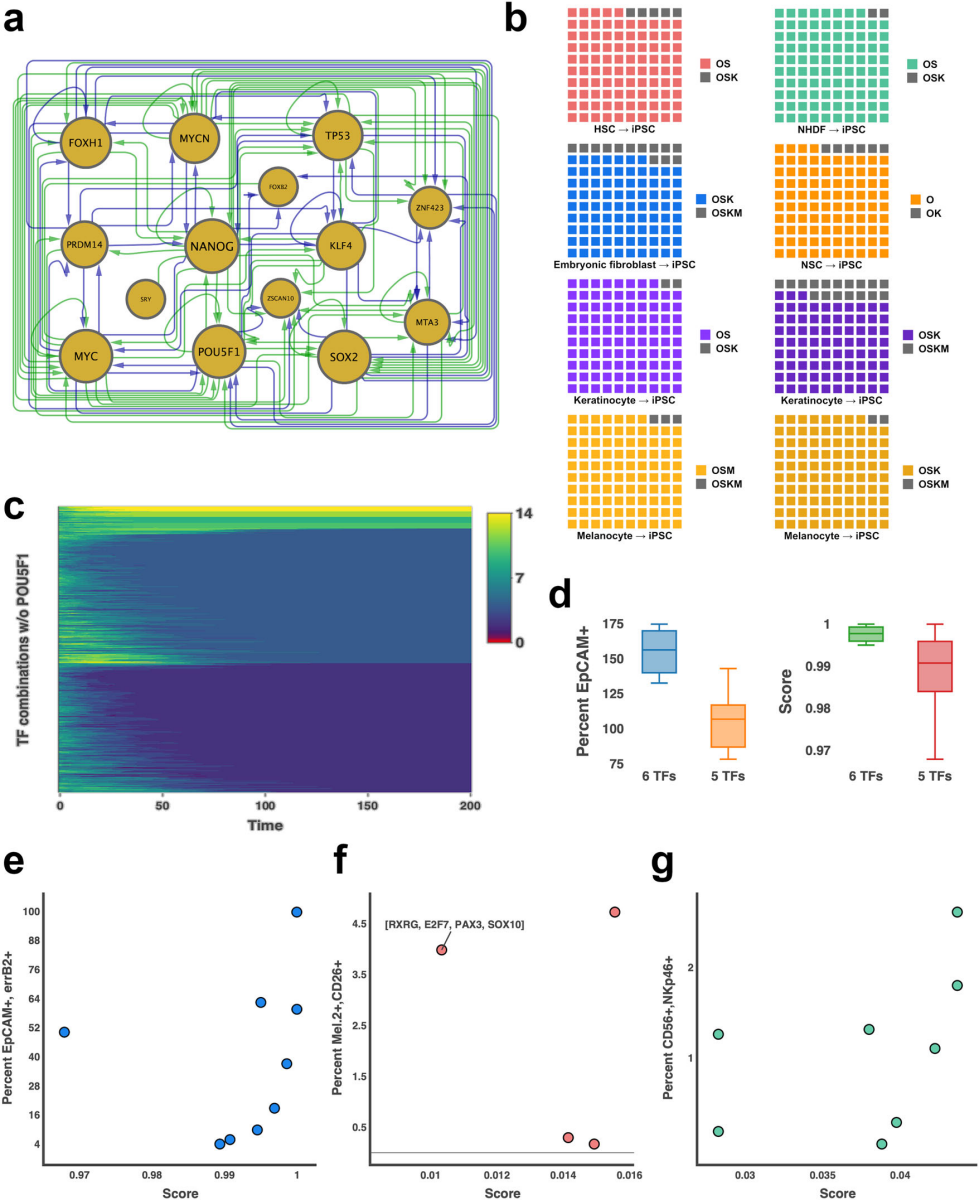
Computational assessment of IRENE’s ability to prioritize IFs. **a** Connected component of the reconstructed GRN of induced pluripotent stem cells. Enhancer and promoter regulation (green) is distinguished from enhancer-only regulation (blue). The size of the nodes (gold) is proportional to the number of regulated TFs. **b** Predicted conversion efficiency for inducing PSCs from hematopoietic stem cells (HSC, red), normal human dermal fibroblasts (NHDF, green), embryonic fibroblasts (blue), neural stem cells (NSC, orange), keratinocytes (purple), and melanocytes (gold). For each conversion, two experimentally validated combinations of IFs were compared. The predicted score of the combinations with the lower experimental efficiency is divided by the predicted score of the combination with the higher experimental efficiency and colored depending on the initial cell type. Each small square in a grid corresponds to 1%. (O = POU5F1, S = SOX2, K = KLF4, M = MYC) **c** Model simulations of 1000 random perturbations of NHDF cells that do not contain POU5F1 for 200 simulation steps. The color code represents the amount of dissimilarity with yellow representing maximum dissimilarity and red depicting perfect agreement. The similarity is measured as the number of expressed TFs in the iPSC network during the simulation. The iPSC network corresponds to dissimilarity of 0 (red) and cannot be induced without POU5F1 being expressed. **d** Comparison of EpCAM-positive cells (left) and predicted scores by IRENE (right) when iPSC differentiation towards mammary epithelial cells is induced with 5- (orange, red) or 6 (blue, green) TF combinations. The median is represented by a solid line within the boxes. The lower and upper bounds of boxes are the first and third quartile, respectively. Whiskers extend to 1.5-times interquartile range or the minimum/maximum value. *n* = 4 (6 TFs) and 5 (5 TFs) independent experiments. e.g., Comparison of scores predicted by IRENE with the percentage of successfully differentiated **e** mammary epithelial cells (blue) **f** melanocytes (red) and **g** NK cells (green).

First, IRENE was employed to reconstruct an iPSC network, which we assessed in terms of its constituent TFs (Fig. 2a). Apparently, except for LIN28A, all known inducers of iPS cells, i.e., NANOG, MYC, POU5F1, SOX2, KLF4, PRDM14, and MYCN, are contained in the network. In addition, the network contains FOXH1, ZNF423, and MTA3, which play diverse roles in the conversion to pluripotent stem cells. For example, FOXH1 significantly enhances iPSC conversion efficiency^40^ and ZNF423 is implicated in the maintenance of pluripotency and self-renewal^8^. In contrast, the functional role of MTA3 in the induction and maintenance of PSCs remains to be investigated. However, TP53 is a constituent of the reconstructed GRN, as well. Even though it is known to diminish iPS cell conversion efficiency^41,42^, TP53 plays an important role in the maintenance of ESCs^41,43^. Due to the dual role of TP53, we examined whether the diminished efficiency of iPSC conversion is reflected in the dynamics of the network. Indeed, combinations including TP53 yield significantly lower scores compared to combinations not containing it (Supplementary Fig. 2, one-sided Wilcoxon–Mann–Whitney test, *p*-value <  1.8e−5). Moreover, the network dynamics underpin the essential role of POU5F1 in the induction of pluripotency, showing that perturbations of fibroblasts without POU5F1 are not capable of activating the complete network (Fig. 2c). In addition, IRENE prioritizes PRDM14 over KLF4, which is consistent with previous reports showing that PRDM14 increases the efficiency of iPS cell conversions^44^ (Fig. 1h).

Supported by the assessment of the iPSC network, we went on to compare the collected dyads of IFs starting from six different initial cell types, i.e., NSCs^45^, HSCs^46^, melanocytes^47^, keratinocytes^48,49^, newborn and adult fibroblasts^50^, and ranked them based on IRENE’s score. Strikingly, IRENE resembled each dyad of combinations correctly and assigned higher scores to combinations with higher efficiency (Fig. 2b).

Finally, since the number of predicted TFs per combination is a user-defined parameter of IRENE, we set out to interrogate the redundancy of predicted TFs in combinations of various sizes. In this regard, we focused on the differentiation of iPSCs into NK-cells, scored all combinations of network TFs of size four, five, and six, respectively, and ranked them based on the predicted scores (Supplementary Fig. 3a). As a result, we observed that the median rank of certain TFs, such as JUN and ELK4, is low, which implies that they predominantly occur in high-ranking combinations of all sizes, whereas others, such as ZNF107 and SP140, mostly occur in low-ranking combinations (Supplementary Fig. 3a). Intrigued by this finding, we explored whether the same trend can be observed for high-ranking combinations as a whole, i.e. whether high-ranking combinations of size *k* are subsumed in high-ranking combinations of size *k* + *1*. However, in contrast to single TFs, the addition of a single factor to high scoring combinations does not always lead to new high scoring combinations, which underscores the highly non-linear dynamics imposed by the cooperative and competitive regulation of TFs (Supplementary Fig. 3b).

### Experimental validation of increased conversion efficiency

**Fig. 3:**
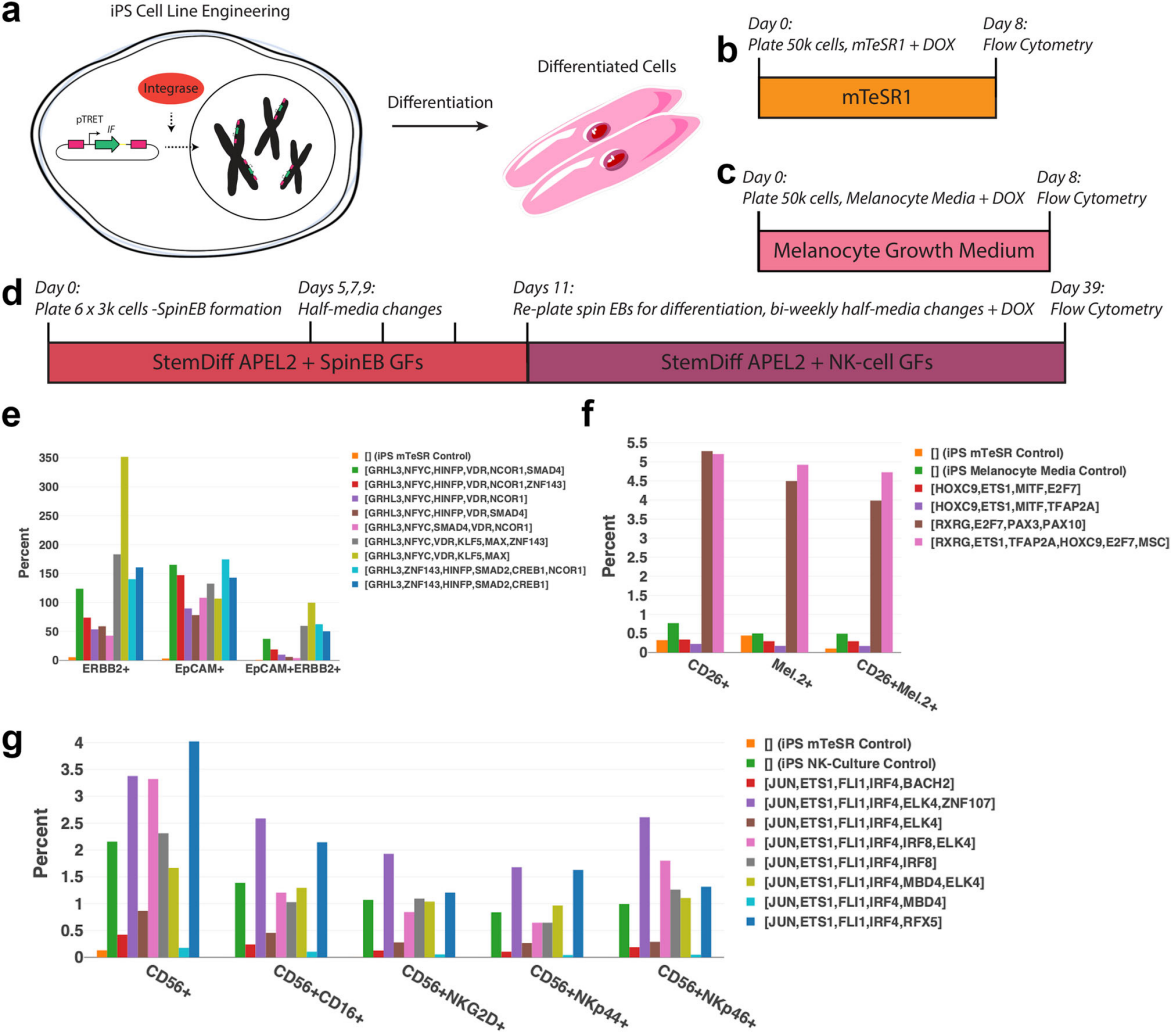
Experimental validation of improved efficiency of cell type conversion. **a** Stable iPS lines for all differentiation experiments were created prior to differentiation via genomic integration to ensure high, stable expression of IFs using the human TFome. **b** Protocol for differentiating human mammary epithelial cells (HMEC) from human iPS cells (hiPSCs). **c** Protocol for differentiating melanocytes from hiPSCs. **d** Protocol for differentiating NK cells from hiPSCs. (GF: growth factor, DOX: doxycycline). **e**–**g** Differentiation efficiency of (**e**). HMECs, **f** melanocytes and **g** NK-cells from hiPSCs for various combinations of IFs generated from IRENE. Efficiency is defined as the number of marker positive/double-positive cells divided by the number of plated cells. *n* = 1 experiment from three pooled biologically independent samples.

To demonstrate IRENE’s ability to predict combinations of IFs, we set out to increase differentiation efficiency by first creating stable iPS lines for all experiments via genomic integration to ensure high, stable expression of IFs using the human TFome (Fig. 3a). We selected the three most commonly used types of protocols: (1) a protocol for differentiating a cell type in the origin media type to demonstrate that the TFs on their own are sufficient for differentiation of a cell type, (2) a differentiation protocol using destination media only to demonstrate that IFs are also effective at differentiating in destination type conditions, and (3) a previously published growth-factor based protocol to show that we can improve differentiation with our identified IFs. We selected three target cell types having an immediate application in therapeutic strategies where conversion efficiency constitutes a major impediment.

For the first, we chose human mammary epithelial cells (HMECs) (Fig. 3b), whose potential in the repopulation of surgically resected mammary tissue has been explored for decades^51^. To date, this requires dissociation of mammary epithelial cells from one tissue environment and subsequent transplantation into another tissue. An efficient in vitro differentiation protocol of mammary epithelial cells would thus overcome this invasive procedure and provides a graft source that can be generated from virtually any patient cells.

For the second, we chose melanocytes (Fig. 3c), which provide a source of cellular grafts to replace damaged cells in the context of vitiligo, an autoimmune disease characterized by the destruction of melanocytes by immune cells, which results in white, unpigmented areas of the skin. To increase accessibility in the clinics and decrease costs, current approaches rely on the use of non-cultured melanocyte grafts, although transplantation of appropriately cultured melanocytes is more efficacious in the re-pigmentation of the skin^52^. Thus, our melanocyte differentiation protocol could serve as a way to increase the accessibility of cultured melanocyte grafts for treating vitiligo in order to achieve more favorable therapeutic outcomes.

For the final, we chose NK-cells (Fig. 3d), whose transplantation from allogeneic donors has been found to have a beneficial effect in the treatment of leukemia after chemotherapy^53^. Although this strategy has been proven useful in achieving a complete remission of the disease in some patients, the transplanted cells were frequently rejected^53^. In this regard, an efficient NK-cell differentiation protocol can substantially benefit the treatment of leukemia by using patient-derived iPSCs, which are expected to be well tolerated.

First, we thought it was important to demonstrate that selected IFs were causing differentiation directly in starting cell type media. To test this, we calculated combinations of TFs for differentiating a cell type without previously documented conversion protocols (mammary epithelial cells) and over-expressed the TFs in iPSCs cultured in stem cell media (mTeSR) (Fig. 3e, Supplementary Fig. 4a). As a result, we observed a high consistency between the experimental and computational ranking of EPCAM and ERBB2 double-positive cells (Fig. 2e). Each of the tested combinations resulted in at least 78.2% EPCAM-positive cells after 8 days, but not necessarily a mammary subtype. In addition, more over-expressed TFs lead to a significant increase in converted epithelial cells (Fig. 2d; Wilcoxon test *p*-value: 0.03). One combination, however, ([GRHL3, NFYC, VDR, KLF5, MAX]), appeared to shift the population double-positive for a large percentage of cells (~99%), compared to the number of seeded cells. To corroborate the induction of these cells, we performed RNA-seq experiments of the initial iPSC and converted cell populations. Comparison between the individual samples with iPSCs confirms the elevated expression of a larger set of mammary epithelial marker genes (Supplementary Fig. 5). In addition, a comparison of network TF expression of the converted cells and iPSCs shows that the over-expression of a small number of TFs was sufficient to induce these TFs in almost all combinations, which supports the network architecture reconstructed by IRENE (Supplementary Fig. 6). Despite the induction of marker genes and network TFs, we set out to assess the transcriptional similarity to mammary epithelial cells by deconvoluting the RNA-seq samples of iPSCs, converted cells, and a gold-standard mammary epithelial cell line (Supplementary Fig. 7). For that, we employed CybersortX^54^, a computational method for detecting the proportion of cell types present in an RNA-seq sample within a single-cell RNA-seq reference dataset. Based on a reference dataset assembled from human breast tumor tissue^55^ and iPSCs^56^, we found up to 14% of the converted cells to possess a mammary epithelial cell type whereas the remaining cells are largely possessing an iPSC phenotype. (Supplementary Fig. 7a). Intriguingly, we employed a HMEC line as a positive control and found only 23% of these cells to possess an epithelial transcriptional phenotype, suggesting a closer resemblance of the converted cells to the positive control than expected from the predicted fraction of epithelial cells. However, we speculate that longer differentiation or differentiation in a mammary-epithelial cell-specific media could result in a more holistic differentiation of the population and, thus, a more pronounced increase in the expression of marker genes and network TFs.

Next, we wanted to determine if IFs selected by IRENE could improve differentiation efficiency when placing cells of the starting type into media of the destination cell type as opposed to the starting cell type (Fig. 3f, Supplementary Fig. 4c). For this experiment, we differentiated iPSCs to melanocytes in melanocyte media with and without TF over-expression. We found that while destination media was sufficient to partially differentiate iPSCs to melanocytes, two of four TF combinations were able to considerably increase the efficiency of differentiation by more than 900% of Mel.2-CD26 double-positive cells (medium alone: 0.49%; TFs: 4.7%) (Fig. 2f). Notably, the lowest ranking combination ([RXRG, PAX3, SOX10, E2F7]) resulted in the second-highest efficiency, only superseded by the combination [RXRG, ETS1, TFAP2A, HOXC9, E2F7, MSC] (Fig. 2f). We suspect that this effect is due to the composition of the growth medium and that it can activate RXRG with retinoic acid, if it is expressed. Indeed, retinoid acid, through RXR activation, is a well-known inducer of melanogenesis^57^. Similar to the case of mammary epithelial cells, RNA-seq confirms the expression of melanocyte marker genes and network TFs, especially for combinations increasing the efficiency (Supplementary Figs. 8, 9). Moreover, deconvolution of the converted cell RNA-seq samples, using a single-cell reference dataset composed of iPSCs^56^ as well as neonatal and adult skin samples enriched for melanocytes^58^, shows up to 93% of successfully converted cells that do not possess an iPSC phenotype anymore (Supplementary Fig. 7b).

Finally, we sought to determine if IRENE could produce combinations of IFs that could increase the conversion efficiency of established differentiation protocols. To test this, we performed NK-cell differentiation using an established differentiation protocol^59^ and measured if the related cellular markers were more prominently differentiated in iPSC lines with over-expressed TFs than a control iPS cell line (Fig. 3g, Supplementary Fig. 4b). Again, we found a high consistency between the experimental and computational ranking of CD56 + NKp46+ double-positive cells (Fig. 2g). In particular, five of eight iPSC lines with combinations of IFs over-expressed after spin-EB differentiation ([JUN, ETS1, FLI1, IRF4, ELK4, ZNF107], [JUN, ETS1, FLI1, IRF4, IRF8, ELK4], [JUN, ETS1, FLI1, IRF4, IRF8], [JUN, ETS1, FLI1, IRF4, MBD4, ELK4] and [JUN, ETS1, FLI1, IRF4, RFX5]) increased the number of CD56 + NKp46 NK-cells by up to 250% compared to the line without IFs, yielding an efficiency of 2.6% with respect to double-positive cells (Fig. 3g). Furthermore, these cell lines expressed a greater percentage of other mature NK-cell markers (Fig. 3g), indicating that not only were more NK-cells produced, but that the cells that were produced were more mature than the iPSC control line. This finding is corroborated by corresponding RNA-sequencing analysis (Supplementary Figs. 10, 11). Except for one combination ([JUN, ELK4, ETS1, FLI1, IRF4]), all combinations induce the expression of NK-cell marker genes and network TFs. This is consistent with the fact that this combination only results in an efficiency of 0.28%, which is lower than the bona fide NK differentiation protocol alone (Fig. 2g). Moreover, deconvolution of converted cell RNA-seq samples using a single-cell reference dataset composed of peripheral blood mononuclear cells^60^ and iPSCs^56^ further underscores the possession of an NK-cell phenotype for most combinations (Supplementary Fig. 7c). In particular, except for one cell line converted with the IF combination [JUN, ETS1, FLI1, IRF4, MBD4], between 16 and 30% of converted cells in each sample are predicted to be NK cells.

## Discussion

The often low efficiency of cellular conversions constitutes a major obstacle in advancing the development of new cell transplantation and gene therapies. Great efforts have been devoted to increasing cell conversion efficiency by employing new experimental techniques for delivering IFs^61–65^ and, in some cases, by developing computational methods for predicting combinations of IFs in specific cellular systems^16–21^. However, none of these approaches alone could systematically address this prevailing issue. Here, we introduced a computer-guided design tool that combines the first computational framework for prioritizing more efficient IFs of cellular conversions, called IRENE, and an experimental setup exploiting the piggyBac transposase to overcome the limitations of viral vector gene delivery.

In particular, IRENE is based on a general strategy for increasing the efficiency of cellular conversions by systematically integrating and making use of transcriptomic and epigenetic profiles. The foundation of IRENE is the reconstruction of cell-type-specific GRNs by integrating chromatin accessibility, histone modifications, TF ChIP-seq, enhancer-promoter interactions, PPIs, and transcriptomic datasets, which allowed the implementation of a model that accounts for the stochastic nature of cellular conversions^10^. The strategy proposed by IRENE for prioritizing more efficient IFs minimizes not only the transcriptional differences between the initial and target cell types but also accounts for the amount of epigenetic restructuring needed during the conversion process, which is a key determinant of conversion efficiency^66^.

As previously described, IRENE reconstructs GRNs based on transcriptional and epigenetic landscapes to predict IFs whose over-expression increases the probability of inducing the target cell type. Moreover, computational over-expression of the predicted IFs has to lead to the satisfaction of all reconstructed logic rules of a network after simulation. It is worth noting that the reconstructed logic rules are static and do not change during simulation, which implies that the predicted IFs for a target cell type requiring the binding of protein complexes in active enhancer or promoter regions have to contain all TFs forming these complexes unless they can be transiently activated. One such example is iPSCs, in which POU5F1 alone or as part of a complex occupies all active regulatory regions. Thus, POU5F1 has been determined to be indispensable for cellular reprogramming, which has long been believed to be true^67^. Nonetheless, recent experiments demonstrated that viable iPSCs can be generated with SOX2, KLF4, and MYC (SKM) alone^68^. Since IRENE used transcriptional and epigenetic profiles of PSCs induced by POU5F1, SOX2, KLF4, and MYC (PSKM), further research is required to assess differences in binding events, active TFs as well as active regulatory regions underlying the reconstructed networks of PSCs induced by SKM and PSKM, respectively. However, to date, no DNase-seq and H3K27ac ChIP-seq data of SKM-based iPSCs have been generated, which currently prevents such an assessment.

Further, predicted IFs by IRENE were over-expressed using piggyBac-integrable TF-over-expression cassettes to overcome the main limitations of current viral vector-based protocols. First, piggyBac can integrate up to 100 kb sections of DNA into the genome^69,70^. In combination with the human TFome^26^, the first collection of more than 1500 TF constructs, virtually any number of predicted IFs can be delivered, thus, overcoming limited carrying capacity. Second, the prevailing issue of genetic silencing is mitigated because piggyBac integrates TF-over-expression cassettes many times (≥40 copies) under recommended nucleofection conditions. Finally, piggyBac enables high-throughput cellular conversions due to its demonstrated low conversion time, which is highly instrumental for testing new gene therapies^71^.

We demonstrated through experimental validation that our computer-guided design tool is applicable to various protocols and substantially increased efficiency in most tested cases. A significant consistency of the rankings of predicted and experimental efficiency has been obtained in the conversion to mammary epithelial and natural killer cells, which proves IRENE’s ability to prioritize more efficient combinations of IFs. For assessing the efficiency of cellular conversions, we adopted the commonly employed formulation, which used the number of starting cells plated and final double-positive cells observed. Although this metric confirmed efficiencies predicted by IRENE, this calculation is inherently unable to account for cell death, proliferation rates, and cells lost during dissociation and washes. Thus, a future improved metric would likely require sophisticated automation for tracking the fate of each cell that divides over the duration of a differentiation protocol. We believe that a significant consistency would be obtained for melanocyte differentiation, if more combinations are tested. In addition, we showed that our tool can be readily applied to an existing protocol of NK cell differentiation and increased the efficiency by 900% compared to existing protocols. Nonetheless, some combinations yielded only a low percentage of CD56 + NKp46+ double-positive cells. Since the predictions have been performed using iPSCs as the initial cell type instead of hematopoietic progenitor cells (HSCs) obtained after 11 days of differentiation, we speculate that low efficiency is due to detrimental regulatory programs established during HSC differentiation, as exemplified by BACH2^72^. Importantly, although experimental validation was performed in the context of directed cellular differentiation, the consistent ranking of IFs for cellular reprogramming towards iPSCs and the high accuracy of recovered IFs in previously established protocols strongly suggests that IRENE could aid in increasing the efficiency of conversions between somatic cells.

In addition to computational methods, several wet-lab approaches have been conceived for predicting efficient IFs of cellular conversions. For instance, a recent study demonstrated that transdifferentiation efficiency can be substantially increased by inducing cells with hyperproliferative and hypertranscribing properties after overexpression of IFs^73^. In contrast, the efficiency of directed differentiation protocols is partly determined by the cell cycle and can be increased through its targeted inhibition. Moreover, the overexpression of IFs using small molecules successfully increased the efficiency of cellular conversions in various cell types^74^. However, the identification of these small molecules requires large amounts of resources and is laborious. Another approach for increasing conversion efficiency is the homogenization of the initial cell source through cell enrichment. This technique has been successfully employed, for instance, to increase the conversion efficiency of cellular reprogramming^75^ as well as the differentiation of monocytes into dendritic cells^76^. In general, the aforementioned approaches have in common that they require knowledge about the IFs inducing the desired cell type. Consequently, we expect that these wet-lab approaches for increasing cellular conversion efficiency are well complemented by the predictions of IRENE.

To our knowledge, our computer-guided tool for designing cellular conversions employs the first computational method that systematically identifies more efficient IFs. Altogether, this tool offers an accurate and efficient method for using TFs in direct cell-type conversion and is expected to enhance the production of cell sources readily usable in therapeutic applications, such as cell transplantation and gene therapies.

## Methods

### Cloning of TF cassettes for cell type conversion

TFs were cloned into a plasmid in between flanking piggyBac integrase regions. Plasmids were part of the Human TFome collection and were cloned with Gateway LR cloning from compatible donor plasmids and did not require primers for amplification. All plasmids are available on the AddGene TFome collection. Upon nucleofection with the piggyBac transposase, DNA between these regions is integrated randomly into the genome. The exact number of integration events was not directly determined, but is a function of DNA quantity upon nucleofection. Upstream of the TF cassettes is a DOX-inducible promoter (pTRET) to activate TF-overexpression in the presence of doxycycline in the media. All plasmids and plasmid maps will be made available on Addgene.

### Creation of cell lines

All differentiating cell lines were performed on reprogrammed PGP1 fibroblasts (https://www.coriell.org/0/Sections/Search/Sample_Detail.aspx?Ref=GM23338&Product=CC) using the Sendai-reprogramming-factor virus. PGP1 iPS cells were expanded and nucleofected with P3 Primary cell 4D Nuceleofection kits with pulse code CB150 using 2 μg of total DNA for 800,000 cells [Lonza]. Cells were plated onto Matrigel-cotated plates [Corning] with ROCK-inhibitor [Millipore] and selected with puromycin [Sigma]. Stable cell lines were expanded over several passages using TrypLE [Gibco] in mTeSR [StemCell Technologies] and frozen in mFReSR [StemCell Technologies]. PGP1 cell lines were modified to incorporate TF over-expression cassettes into the genome to create cell lines. The following cell lines were created (ex: “Cell line name [TF1, TF2,…]”): NK 5.1 [JUN,ELK4,ETS1,FLI1,IRF4]; NK 5.2 [JUN,ETS1,FLI1,IRF4,MBD4]; NK 5.3 [JUN,ETS1,FLI1,IRF4,RFX5]; NK 5.4 [JUN,ETS1,FLI1,IRF4,IRF8]; NK 5.5 [JUN,BACH2,ETS1,FLI1,IRF4]; NK 6.1

[JUN,ELK4,ETS1,FLI1,IRF4,IRF8]; NK 6.2 [JUN,ELK4,ETS1,FLI1,IRF4,ZNF107]; NK 6.4

[JUN,ELK4,ETS1,FLI1,IRF4,MBD4]; Mel L [E2F7,SOX10,PAX3,RXRG]; Mel H1 [MITF,ETS1,HOXC9,TFAP2A]; Mel H2 [E2F7,ETS1,HOXC9,TFAP2A]; Mel 5.1 [RXRG,ETS1,SOX10,MITF,TFAP2A]; Mel 6.1

[RXRG,ETS1,HOXC9,E2F7,TFAP2A,MSC]; HMEC 5.1 [GRHL3,NFYC,VDR,KLF5,MAX]; HMEC 5.2

[GRHL3,NFYC,VDR,NCOR1,HINFP]; HMEC 5.3 [GRHL3,NFYC,VDR,SMAD4,HINFP]; HMEC 5.4

[GRHL3,NFYC,VDR,NCOR1,SMAD4]; HMEC 5.5 [GRHL3,HINFP,ZNF143,SMAD2,CREB1]; HMEC 6.1

[GRHL3,NFYC,ZNF143,VDR,KLF5,MAX]; HMEC 6.2 [GRHL3,NFYC,VDR,NCOR1,SMAD4,HINFP]; HMEC 6.3

[GRHL3,NFYC,VDR,NCOR1,HINFP,ZNF143]; HMEC 6.4 [GRHL3,HINFP,ZNF143,SMAD2,CREB1,NCOR1].

### HMEC differentiation

In total, 50,000 hiPSCs were plated on matrigel-coated plates and differentiated with 2 ng/mL doxycycline [Sigma] for 8 days in mTeSR [StemCell Technologies] with full media changes daily.

### Melanocyte differentiation

In total, 50,000 hiPSCs were plated on matrigel-coated plates and differentiated with 2 ng/mL doxycycline [Sigma] for 8 days in Melanocyte Growth Media [Sigma] with full media changes every other day.

### NK-cell differentiation

Six wells of 3000 hiPSCs were plated into uncoated round-bottom plates in 200 μL of StemDiff APEL2 media [StemCell Technologies] with Stem Cell Factor (SCF) (40 ng/mL) [R&D Systems], BMP4 (40 ng/mL) [R&D systems], and VEGF (40 ng/mL) [BioLegend] and spun at 300 g at RT for 5 min. Cells were then incubated at 37 C and 5% CO_2_ for 11 days. Half-media changes were performed on days 5, 7, and 9. On day 11, all six wells of spin EBs were aspirated without disturbing the structure of the EB and plated into a well of a 24-well plate in NK differentiation media consisting of StemDiff APEL2, SCF (20 ng/mL), IL-3 95 (5 ng/mL) (first week only) [R&D Systems], IL-7 (20 ng/mL) [R&D Systems], IL-15 (10 ng/mL) [R&D Systems], and FlT3l (10 ng/mL), [BioLegend]. Half media changes were performed once per week for 4 weeks.

### Flow cytometry

Cells were digested in TrypLE [Gibco] and resuspended in growth media before staining with cell surface markers. The following antibodies were used for analysis: [HMEC: ERB2-APC-Vio-777 (10 μL/test), EpCAM-PE-Cy7 (5 μL/test)]; [NK-cells: CD56-APC (5 μL/test), CD16-PerCP-Cy5.5 (5 μL/test), NKp44-PE (5 μL/test), NKp46-PE-Cy7 (5 μL/test), NKG2D-FITC (5 μL/test)]; [Melanocytes: CD26-PerCP-Cy5.5 (5 μL/test), Mel.2-anti mouse IGg1 (1 μg/mL), ms IGg1-PE (20 μL/test)]. Cells were analyzed on a BD LSR Fortessa Analyzer. We measured 3 biological replicates and for at least 1000 cells. Cytometry results were analyzed using the flowCore R package v1.52.1 and related packages.

### RNA Sequencing

100k or fewer cells were digested with TrypLE [Gibco] and resuspended in TRIzol LS Reagent [Invitrogen] for lysis. The RNA was purified using a Direc-zol RNA MicroPrep Kit [Zymo]. Library preparation was performed with a SMARTer Seq v2 Pico Mammalian Input kit [TAKARA Bio]. NGS was performed using Illumina NovaSeq technology for 115 cycles.

### Identification of identity TFs

A background gene expression distribution of each TF was defined by 7600 different samples in Recount2^77^ (Supplementary Data 3). All samples from The Cancer Genome Atlas (TCGA) and those containing the terms “cancer”, “disease”, and “single cell” in the title or description of their Gene Expression Omnibus (GEO)^78^ entry were excluded prior to the analysis. TFs in a query sample were subsequently ranked based on the specificity of their expression using a modified version of the method proposed by D’Alessio et al.^16^. The approach consists of three steps. First, gene expression profiles in the background are excluded that are correlated to the query sample. A Pearson correlation coefficient of 0.75 was selected as a threshold, by maximizing the F1 score of distinguishing ESC from non-ESC samples in the background distribution (Supplementary Fig. 12). Second, for each TF, an idealized probability distribution, which contains ‘1’ in place of the considered sample and ‘0’ otherwise, and a query probability distribution, containing the normalized expression of the TF in all samples, is created. Finally, the Jensen-Shannon divergence (JSD) between the ideal and background distribution is computed. The 10 TFs having the highest JSD value are selected as identity TFs.

### Reconstruction of cell-type-specific core GRNs

GRN reconstruction follows a three-step approach. First, every gene is classified into being active or inactive based on its expression value using RefBool^79^ with Matlab 2018a (© Mathworks), testing the null hypothesis that a gene is inactive. *P*-values of <0.1 were considered significant.

Second, active proximal and distal regulatory regions are identified for every active TF. Promoters are defined based on the Ensembl promoter annotation from the Eukaryotic Promoter Database^80^ (accessed 23 March 2018) and restricted to 1500 bp upstream and 500 bp downstream. Promoter regions are deemed active in a given cell type if it overlaps with at least one H3K4me3 peak. Enhancers of active TFs are defined by the GeneHancer database^27^ (accessed 6 April 2018). Enhancers are deemed active if they overlap with at least one H3K27ac peak and truncated to the peak region. Inactive enhancer regions are discarded.

Finally, TF binding events are identified in active promoter and enhancer regions by overlaying TF ChIP-Seq peaks from ChIP Atlas^30^, regardless of the cell type they were profiled in. Every binding event sharing one base pair with an active region constitutes a potential regulatory interaction. Interactions are filtered by cell-type-specific DNase-Seq peaks, such that all remaining interactions are within accessible chromatin regions.

Using this approach, a GRN scaffold is constructed among all TFs and subsequently restricted to identity TFs and co-factors. Co-factors are selected based on three conditions. First, only active TFs defined by RefBool are considered. Next, TFs are ranked based on their JSD value and restricted to those whose ranks are significantly lower than their average rank across all samples (z-score ≤ −1.5). Finally, co-factors must regulate and must be regulated by at least one identity TF. The GRN scaffold is restricted to identity TFs and co-factors, which constitutes the core GRN.

Accessions of the experimental datasets used in this study are provided in Supplementary Data 4. All considered datasets were annotated to genome assembly GRCh38 or converted to GRCh38 by using the CrossMap tool^81^.

### Inference of Boolean logic rules

IRENE infers cooperative and competitive binding by identifying TFs with overlapping ChIP-seq peaks having a PPI reported in iRefIndex^37^. Significant overlap is determined on the basis of positive and negative gold-standard datasets of 755 and 336 PPIs, respectively^82,83^. The average reciprocal overlap of (non-)interacting TF pairs was computed in all cell lines/cell types with available ChIP-seq profiles in ChIP-Atlas^30^. TF pairs are more likely to interact if their peaks overlap by at least 62.43% (Supplementary Fig. 13). All TFs in an active regulatory region are transformed into an undirected graph where an edge represents an overlap of more than 62%. The connected components of this graph are detected using the “clusters”-method of the R “igraph”-library (version 1.2.2)^84^.

TFs in a cluster are connected by a Boolean AND-gate while all others are connected by an OR-gate. Active enhancer and promoter regions are combined by forcing the regulation of at least one enhancer, thus connecting multiple enhancers by an OR-gate, and the promoter.

### Prediction of efficient combinations of IFs

IRENE computes a surrogate measure of cellular conversion efficiency by assessing the probability that the over-expression of a set of TFs eventually activates the complete core GRN. The measure is composed of a transcriptional and an epigenetic score.

For the transcriptional score, the RNA-seq profiles of the initial cell type are processed and discretized as described before. Over-expression of TFs is performed in the discrete space, switching the expression value from ‘0’ to ‘1’. A prior distribution over all GRN states is computed describing the probability of the initial cell type to be in each network state after applying over-expression of TFs. The probability for each TF to be active or inactive is defined as the probability of observing a lower or greater expression value in the background distribution of RefBool^79^, respectively. The probability of being in a certain network state is then defined as the product of the probabilities of being in the individual TF states. Finally, the model checker PRISM v4.4^85^ is employed to compute the distance of all network states that eventually activate the whole network (Supplementary Note 2). The transcriptional score is defined as the average distance to the desired network state in which every TF is actively weighted by the prior distribution.

For the epigenetic score, IRENE computes the fraction of common active regulatory elements after TF over-expression. This fraction is set to one for over-expressed TFs, thus bypassing the need for remodeling. The product of the fraction of common enhancers after applying a perturbation serves as the epigenetic score.

Finally, the arithmetic mean of the epigenetic and transcriptional scores constitutes IRENE’s surrogate measure of efficiency.

Importantly, IRENE requires the specification of a user-defined number of TFs included in the combinations to allow for accounting for different experimental setups.

### GO enrichment

GO enrichment was performed using the WebGestaltR R-package v0.4.2 with R version 3.6.1. All network TFs were queried against categories defined in the “biological process” database that have at least 10 and at most 200 annotated genes and restricted to human TFs from AnimalTFDB v3^86^. A false discovery rate less than 0.05 was considered significant.

### Prediction of promoter regulators from DNA-binding predictions

Known motifs for the human Grch38 genome were obtained from the Homer webpage (version 191020) as bed-files and subset to the TFs included in the melanocyte network. The set of all binding sites was subset to network TF promoter regions defined by the Eukaryotic Promoter Database^80^ (restricted to 1500 bp upstream and 500 bp downstream) using the intersectBed program from bedtools^87^ v2.22.1.

### Determining statistical significance of IF prioritization

Concordance of rankings obtained from experimentally measured conversion efficiency and predicted scores from IRENE is assessed by calculating the Euclidean distance between both rankings. Statistical significance was assessed by computing a background distribution of the Euclidean distance between all possible rankings and the experimentally obtained ranking. The *p*-value was defined as the cumulative probability of observing a lower distance than the one obtained from the predicted scores.

### Deconvolution of RNA-seq samples

RNA-seq samples are deconvoluted into individual cell types using CybersortX^54^. First, single-cell SmartSeq2 reference datasets have been collected and normalized to TPM. For each reference dataset, a signature matrix containing 500 to 700 genes per cell type was computed using CybersortX. Only genes having an expression value above 2 TPM were considered and no sub-sampling of cells was performed. RNA-seq counts were transformed to TPM and served as an input for deconvolution. CybersortX was run in “absolute mode” to allow for normalization of the deconvolution results into percentages.

### Reporting summary

Further information on research design is available in the Nature Research Reporting Summary linked to this article.

## Supplementary information

Supplementary Information

Description of Additional Supplementary Files

Supplementary Data 1

Supplementary Data 2

Supplementary Data 3

Supplementary Data 4

Supplementary Data 5

Supplementary Data 6

Reporting Summary

## Data Availability

The RNA-seq data generated in this study can be found in GEO: GSE165961. Accession numbers of transcriptomics datasets used for identifying identity TFs are provided in Supplementary Data 3. Accession numbers of datasets employed for reconstructing gene regulatory networks can be found in Supplementary Data 4. TF ChIP-seq accession numbers for network reconstruction are provided in Supplementary Data 5. Supplementary Data 6 contains pre-computed combinations of IFs for various cellular conversions. Databases used throughout this study are publicly available: Eukaryotic Promoter Database [https://epd.epfl.ch/human/human_database.php?db=human], AnimalTFDB v3 [http://bioinfo.life.hust.edu.cn/static/AnimalTFDB3/download/Homo_sapiens_TF], GeneHancer v4.7 [https://genecards.weizmann.ac.il/geneloc_prev/genehancer.xlsx], Chip Atlas [https://chip-atlas.org/peak_browser], iRefIndex [https://irefindex.vib.be/] and Recount2 [https://jhubiostatistics.shinyapps.io/recount/]. The datasets used for generating single-cell RNA-seq reference samples for bulk RNA-seq deconvolution are publicly available in GEO: iPSC [https://www.ebi.ac.uk/arrayexpress/files/E-MTAB-6819/E-MTAB-6819.processed.1.zip], breast tumor tissue [https://www.ncbi.nlm.nih.gov/geo/query/acc.cgi?acc=GSE118389], PBMCs [https://www.ncbi.nlm.nih.gov/geo/query/acc.cgi?acc=GSE132044] and melanocytes [https://www.ncbi.nlm.nih.gov/geo/query/acc.cgi?acc=GSE151091].

## Code availability

The source code of IRENE is available at https://github.com/saschajung/IRENE.
