# Supplementary material for: A computer-guided design tool to increase the efficiency of cellular conversions
Source: Nat Commun. 2021 Mar 12;12:1659. doi: 10.1038/s41467-021-21801-4 (PMC7954801; doi:10.1038/s41467-021-21801-4)
Supplement: Supplementary file 1 — Supplementary Information [file 41467_2021_21801_MOESM1_ESM.pdf]

Supplementary Notes

Supplementary Note 1: Validation of reconstructed GRNs

To assess the cell-type-specificity of reconstructed core networks, we conducted a comparison with manually curated networks from literature that contain experimentally validated interactions. The set of gold-standard networks is composed of human hepatocytes[1], embryonic stem cells (ESCs)[2] and two cancer cell lines (MCF7 and HepG2)[3], and was compared on the subset of TFs that is present in the reconstructed core GRNs.

In addition, we leveraged cell-type-specific TF ChIP-Seq data to calculate the enrichment of experimentally validated interactions in the reconstructed GRNs. For this purpose, uniformly processed TF ChIP-Seq peaks were gathered from ChIP-Atlas[4]. In order to keep the data consistent between these two resources, all peak files were converted to GRCh38 genome assembly by using the CrossMap tool[5], if they were aligned to a different assembly. ENCODE and GEO accessions of all the considered TF ChIP-Seq experiments are given in Supplementary Data 5. Based on these datasets, two analyses were carried out. First, the fraction of TF interactions in promoter regions was assessed that are validated by a peak in the cell-type-specific ChIP-Seq experiments. For that, only those TFs in the networks were considered for which ChIP-Seq data was available. Second, the number of false positive interactions was quantified by counting the fraction of interactions in promoter regions having no ChIP-Seq peak in the same cell type/line but in another cell type/line.

Supplementary Note 2: Model Checking GRNs with PRISM

Since PRISM is unable to handle Boolean logic rules but evaluates polynomials, IRENE transforms them into equivalent polynomials by the following rules[6]:

Given two TFs A and B in the GRN, the following relations hold.

(i) $\neg A \equiv 1 - A$

(ii) $A \vee B \equiv A + B - A \cdot B$

(iii) $A \wedge B \equiv A \cdot B$

While the second rule states a valid transformation of Boolean rules into polynomials, it is impractical in the presence of multiple TFs with competing or non-overlapping binding sites within regulatory regions. By applying De Morgan's law ($A \vee B \equiv \neg(\neg A \wedge \neg B)$), a fourth rule can be derived that is easily adaptable to multiple TFs:

(iv) $A \vee B \equiv \neg(\neg A \wedge \neg B) \equiv 1 - ((1 - A) \cdot (1 - B))$

Using these transformations, a PRISM model of the DTMC is established in which each TF is a module that can change its state based on the evaluation of the polynomial expressions. During each simulation step of the model, a single TF is selected randomly and its state is updated, i.e. the DTMC obeys an asynchronous updating scheme. Finally, the property that has to be checked by PRISM was stated as "what is the average path length of every state from which eventually all TFs in the network are active". In PRISM syntax this corresponds to "$\mathrm{filter}(\mathrm{print}, \mathrm{R}\ (TF_1 + TF_2 + \cdots + TF_n = n))$". The filter function combined with the 'print' argument returns the path length for all states.

Supplementary Tables

Cell type	TFs in core network	Core TFs in the network	coTFs in the network	Differentially expressed core TFs	Differentially expressed coTFs	coTFs in IF sets
Hepatocytes	15	7	8	7	7	2
Melanocytes	20	10	10	10	9	4
Myoblasts	19	10	9	10	8	1
Neurons	7	2	5	2	5	1
NSCs	14	4	10	4	10	5
Adipocytes	16	2	14	2	14	7

Supplementary Table 1. Related to Figure 1. Differential expression analysis of co-TFs. Assessment of differentially expressed core TFs and their co-factors for six cellular conversions depicted in Figure 1h.

	ESC	Hepatocytes	HepG2	MCF7
TFs in GS	3	6	4	4
GS TFs in reconstructed GRN	3	4	4	2
GS-Interactions	9	13	16	13
Inferred-Interactions	9	12	16	4
Matching	9	8	16	4
Unique-GS	0	5	0	9
Unique-Inferred	0	4	0	0
Inferred-Validated	0	4	0	0
Overall-Validated	100%	70.59%	100%	30.77%
Approach2 Matching	2	6	16	4
Approach2-Inferred	0	3	0	0
Approach2-Validated	0	3	0	0
Approach2-Validated	22.22%	56.25%	100%	30.77%

Supplementary Table 2. Related to Figure 1. Gold standard network comparison with IRENE. Statistics about the network similarity assessment of four gold standard networks. To validate the choice of databases underlying IRENE, the assessment was performed as well as for reconstructed networks using EnhancerAtlas and GTRD for enhancer-gene associations and transcriptional regulatory interactions, respectively.

Supplementary Figures

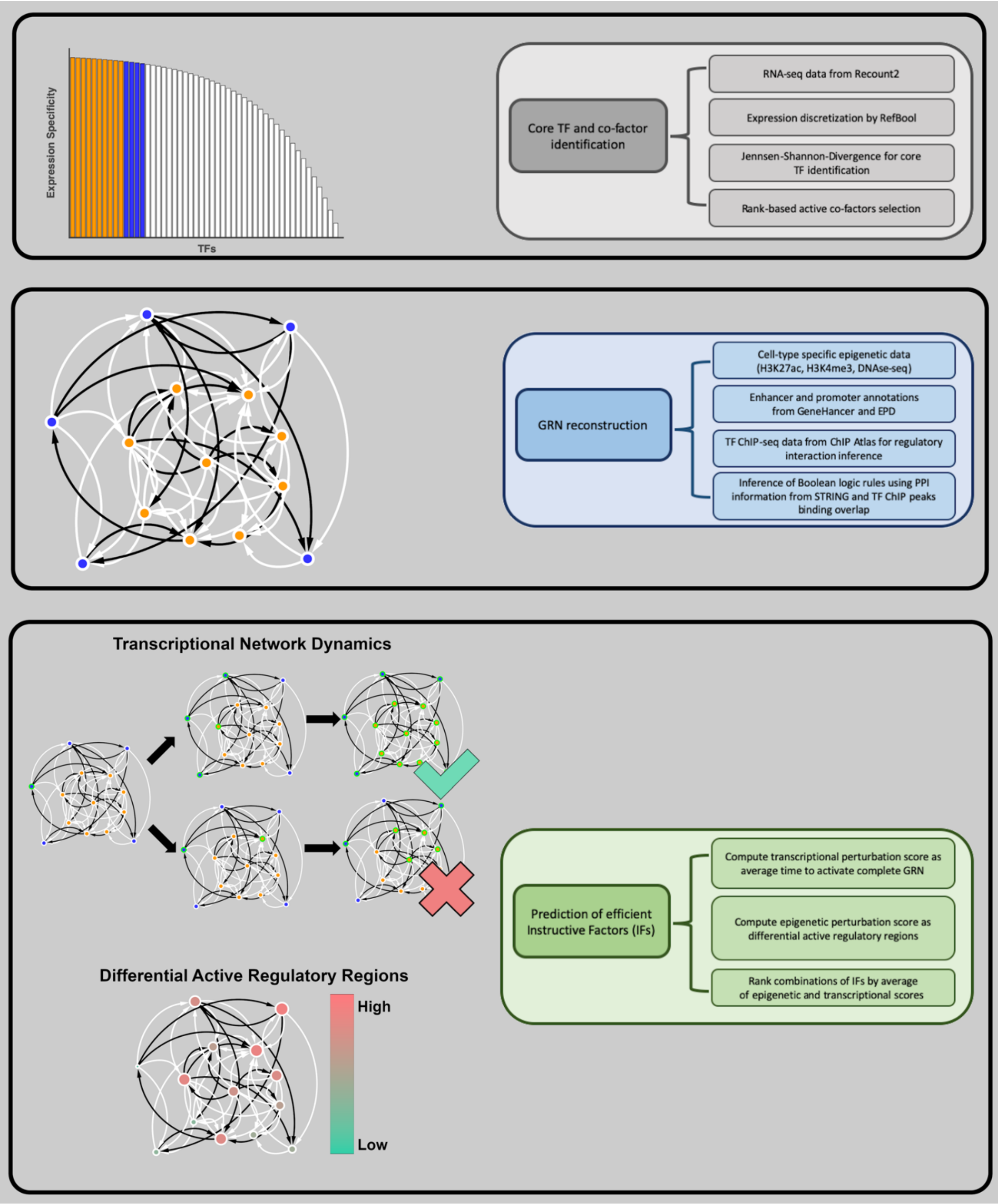

Supplementary Figure 1. Related to Figure 1. Schematic workflow of IRENE. Identity TFs (orange) and co-factors (blue) are identified using Jensen-Shannon-Divergence (top) for network reconstruction. Interactions between these TFs are identified by integrating epigenetic information. Co-Factors that are regulated by at least one identity TF and that regulate at least one identify TF are retained in the network (middle). The probability of TF combinations to activate the complete network is assessed by simulations and the percentage of epigenetic restructuring is quantified. The average of both assessments constitutes the final score of a TF combination

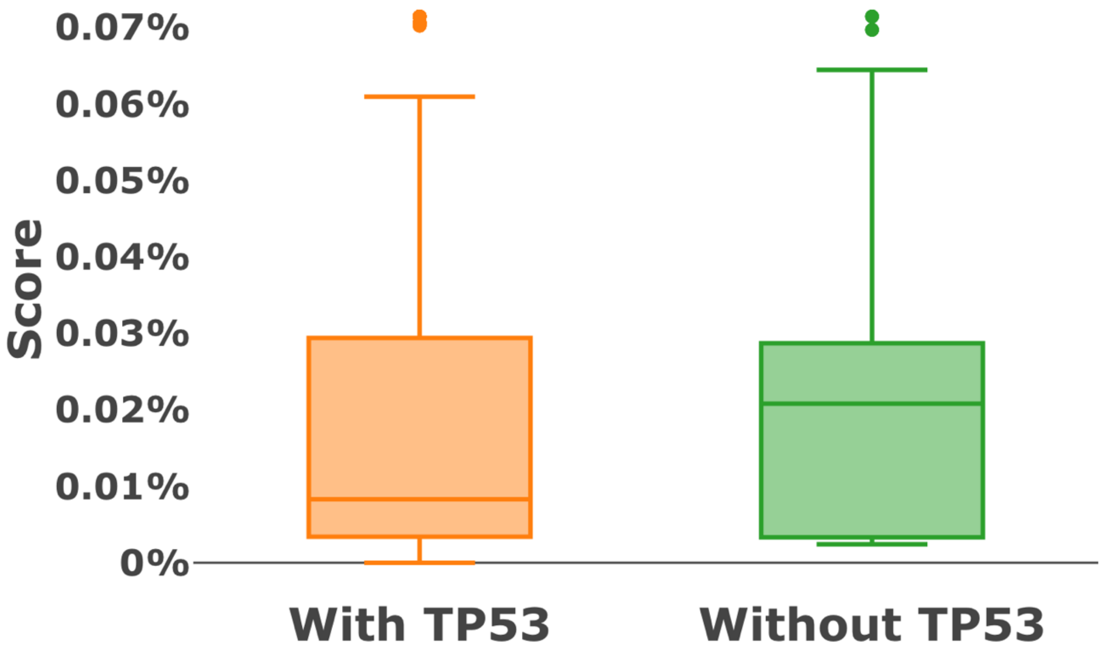

Supplementary Figure 2. Related to Figure 2. Comparison of scores with and without TP53. Scores of IRENE were computed for inducing the human iPSC network with all possible combinations of network TFs. Scores of combinations with (orange) and without (green) TP53. The median scores of combinations without TP53 are higher compared to those combinations with TP53. The ends of the box represent the lower and upper quartiles, while the median is marked by a line inside the box. Whiskers extend to 1.5-times the interquartile range. Dots represent outliers. n = 8191 independent combinations of network TFs with and without TP53, respectively.

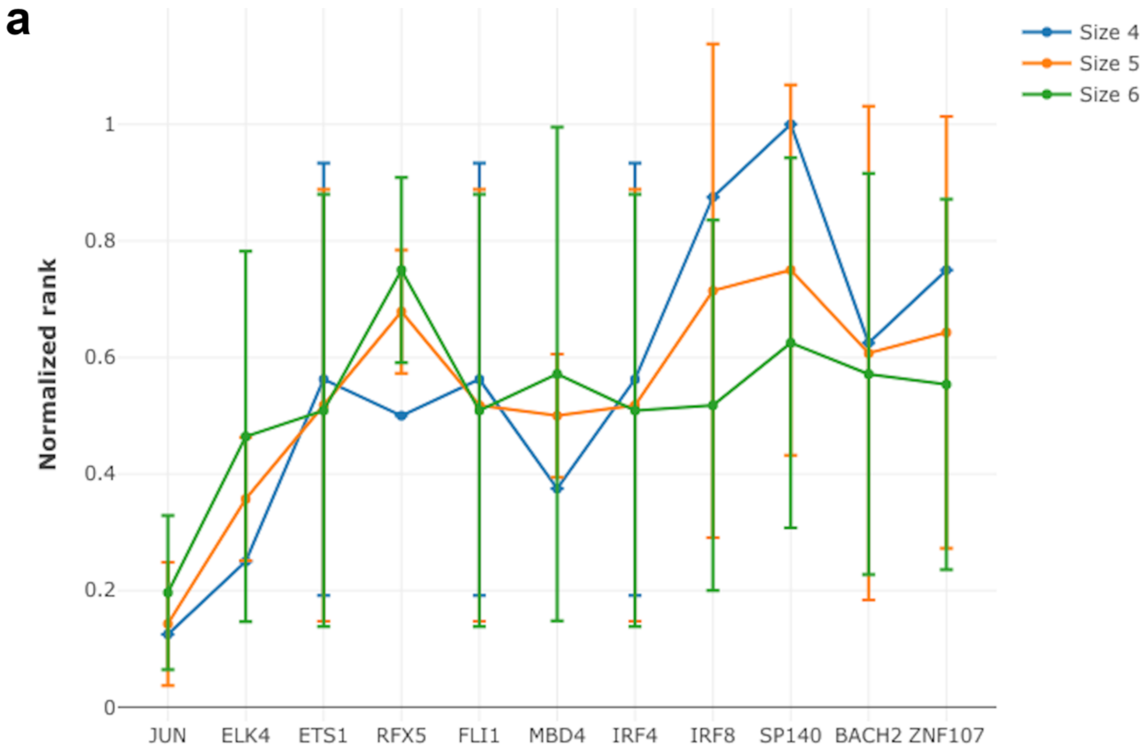

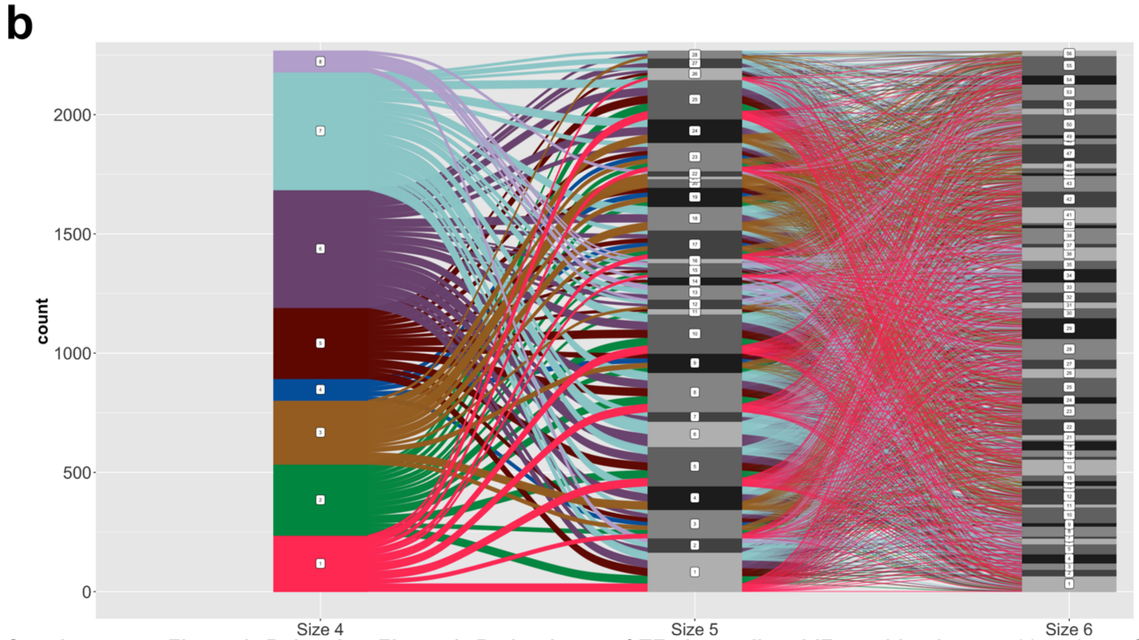

Supplementary Figure 3. Related to Figure 2. Redundancy of TFs in predicted IF combinations. a. Line chart of normalized ranks of combinations including the corresponding TF. Ranks are normalized by the number of predicted combinations of each size that are able to reach the NK cell state. Thus, lower normalized ranks correspond to higher scores and lower ranks. Dots represent the median normalized ranks, error bars extend to +/- median absolute deviation. n = 8 (size 4), 28 (size 5) and 96 (size 6) combinations inducing the NK cell state. **b.** Alluvial plot of predicted TF combinations of various sizes that are able to reach the NK-cell state. Numbers represent the rank with respect to the scores with lower ranks representing higher predicted scores. Colors represent individual combinations of size 4. A flow from combination i of size j to a combination k of size j+1 is established if the i is a subset of k.

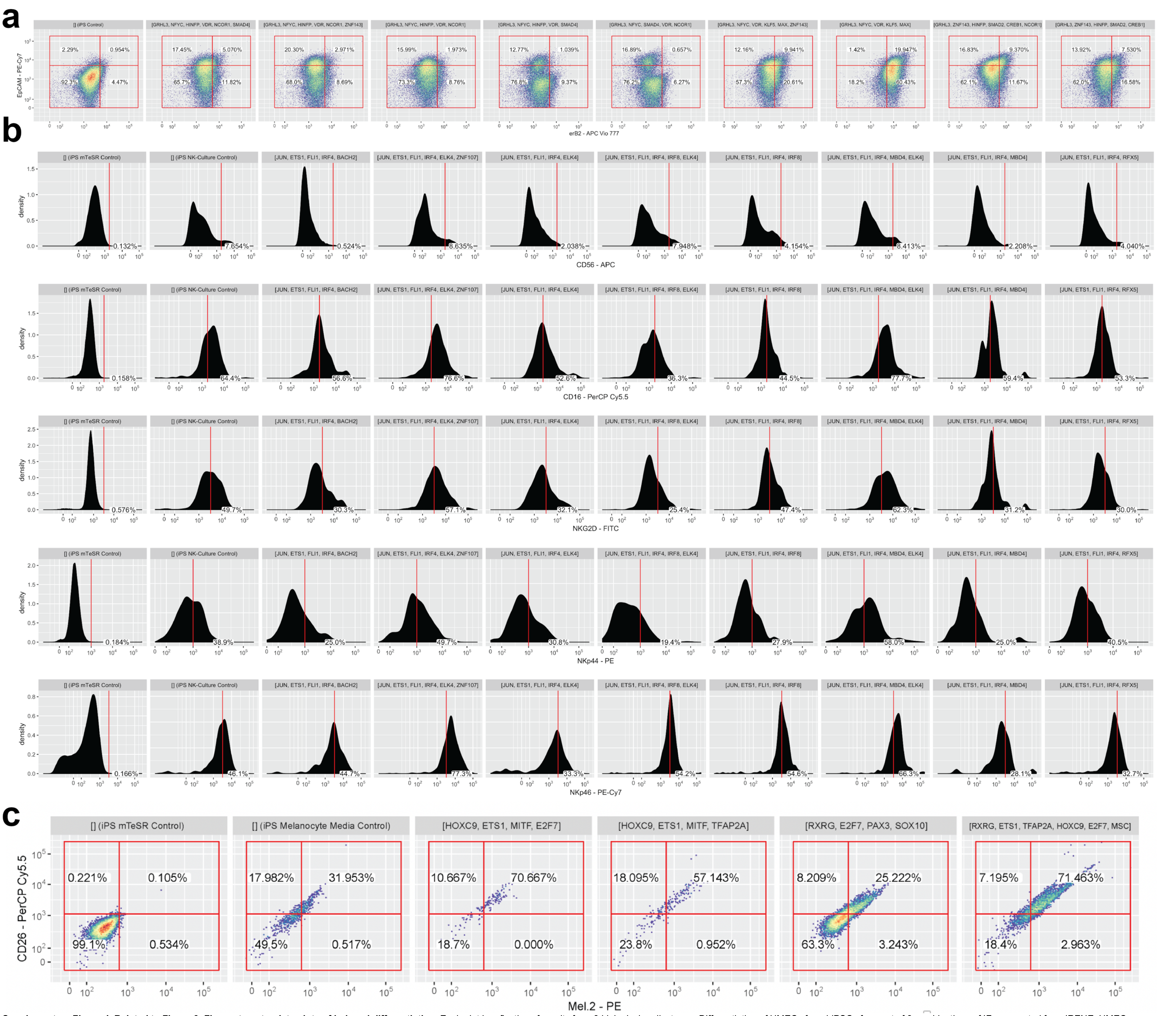

Supplementary Figure 4. Related to Figure 3. Flow cytometry data plots of induced differentiation. Each plot is reflective of results from 3 biological replicates. **a.** Differentiation of HMECs from hiPSCs for a set of 9 combinations of IFs generated from IRENE. HMECs are identified by cells double-positive for EpCAM (epithelial marker) and ERB2 (mammary marker) relative to hiPSCs. **b.** Differentiation of NK-cells from hiPSCs for a set of 9 combinations of TFs generated from IRENE. NK cells are identified by cells positive for CD56 and NKp46. Far left panel is an hiPSC control grown in mTeSR media, second to the left are iPSCs without TFs differentiated as per published protocol'. CD56 positive cells are pulled out from and evaluated for other NK-cell markers (CD16, NKG2D, NKp44 and NKp46). **c.** Differentiation of melanocytes from hiPSCs for a set of 5 combinations of TFs generated from IRENE. Melanocytes are identified by cells double-positive for Mel.2 and CD26

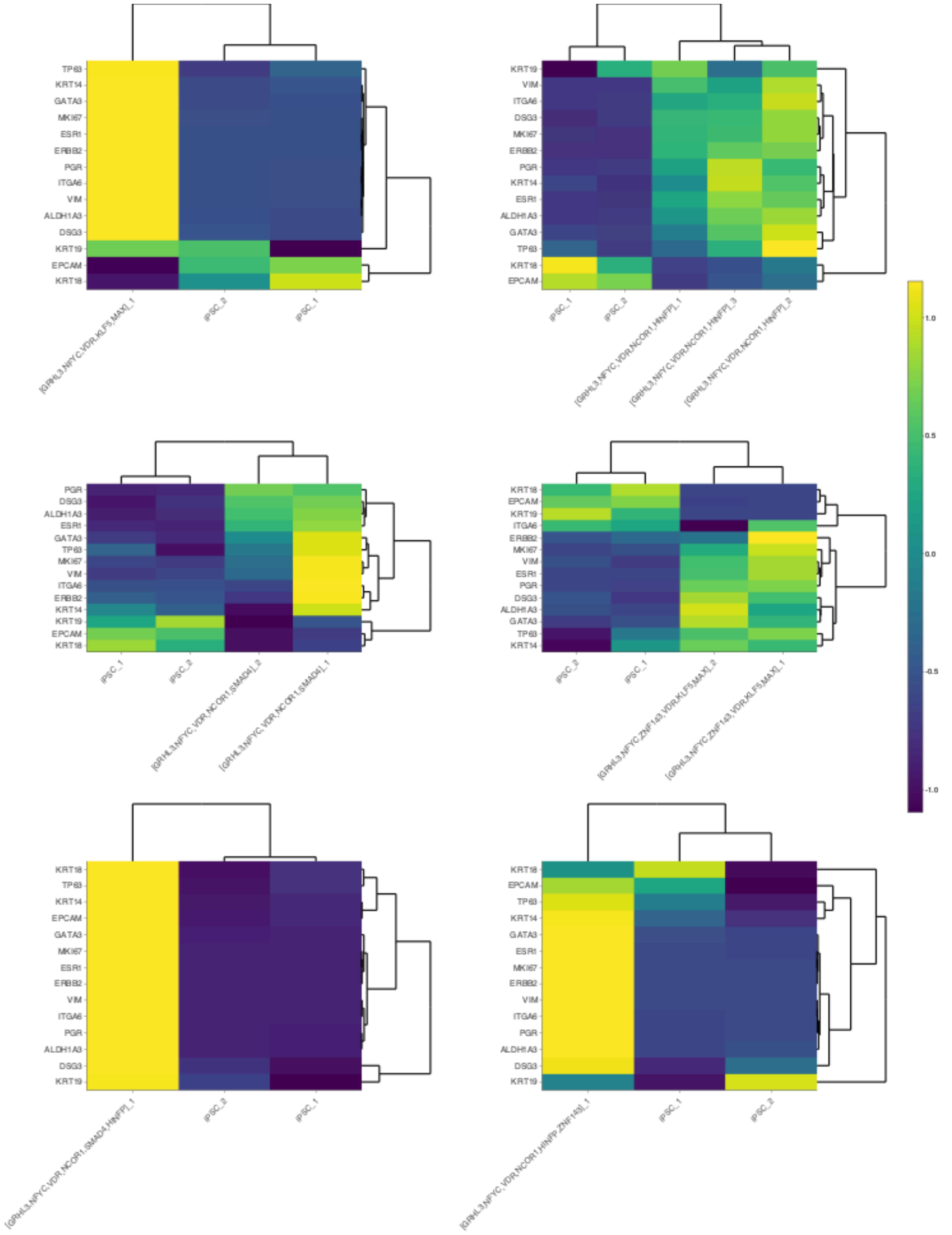

Supplementary Figure 5. Related to Figure 3. HMEC marker gene expression. Gene expression from RNA-seq samples of converted cell populations are compared to iPSCs, the initial cell type of the conversion. Heatmaps show scaled expression values of collected marker genes for up- (yellow) and down-regulated (blue) genes. Combinations of IFs are denoted in brackets. Scaling was performed row-wise. Multiple replicates from the same cell population are marked by an underscore followed by a sequential number. Only samples having more than 5 million reads were considered for this assessment.

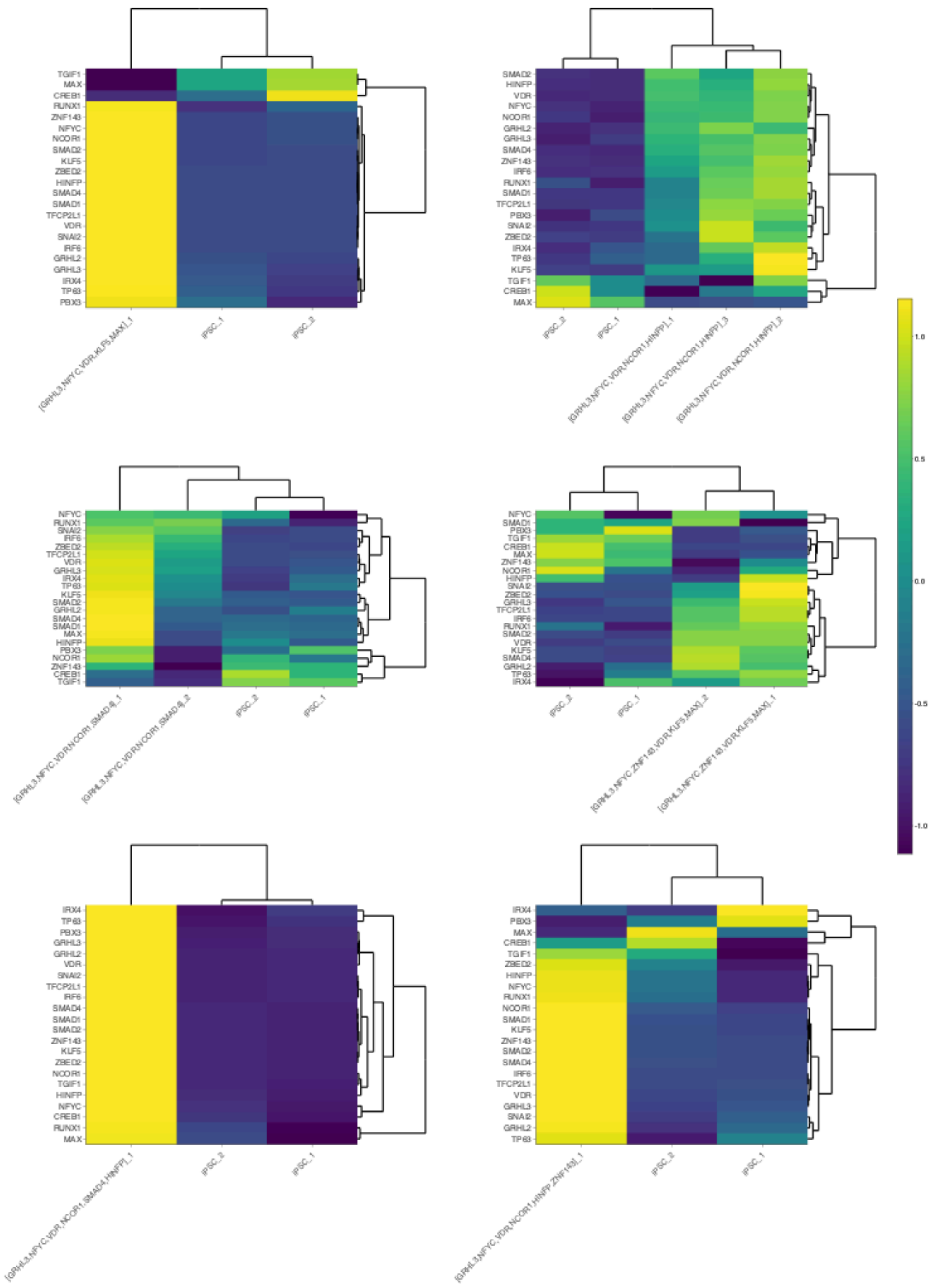

Supplementary Figure 6. Related to Figure 3. HMEC network TF gene expression. Gene expression from RNA-seq samples of converted cell populations are compared to iPSCs, the initial cell type of the conversion. Heatmaps show scaled expression values of TFs present in the mammary epithelial cell network reconstructed by IRENE for up- (yellow) and down-regulated (blue) genes. Combinations of IFs are denoted in brackets. Scaling was performed row-wise. Multiple replicates from the same cell population are marked by an underscore followed by a sequential number. Only samples having more than 5 million reads were considered for this assessment.

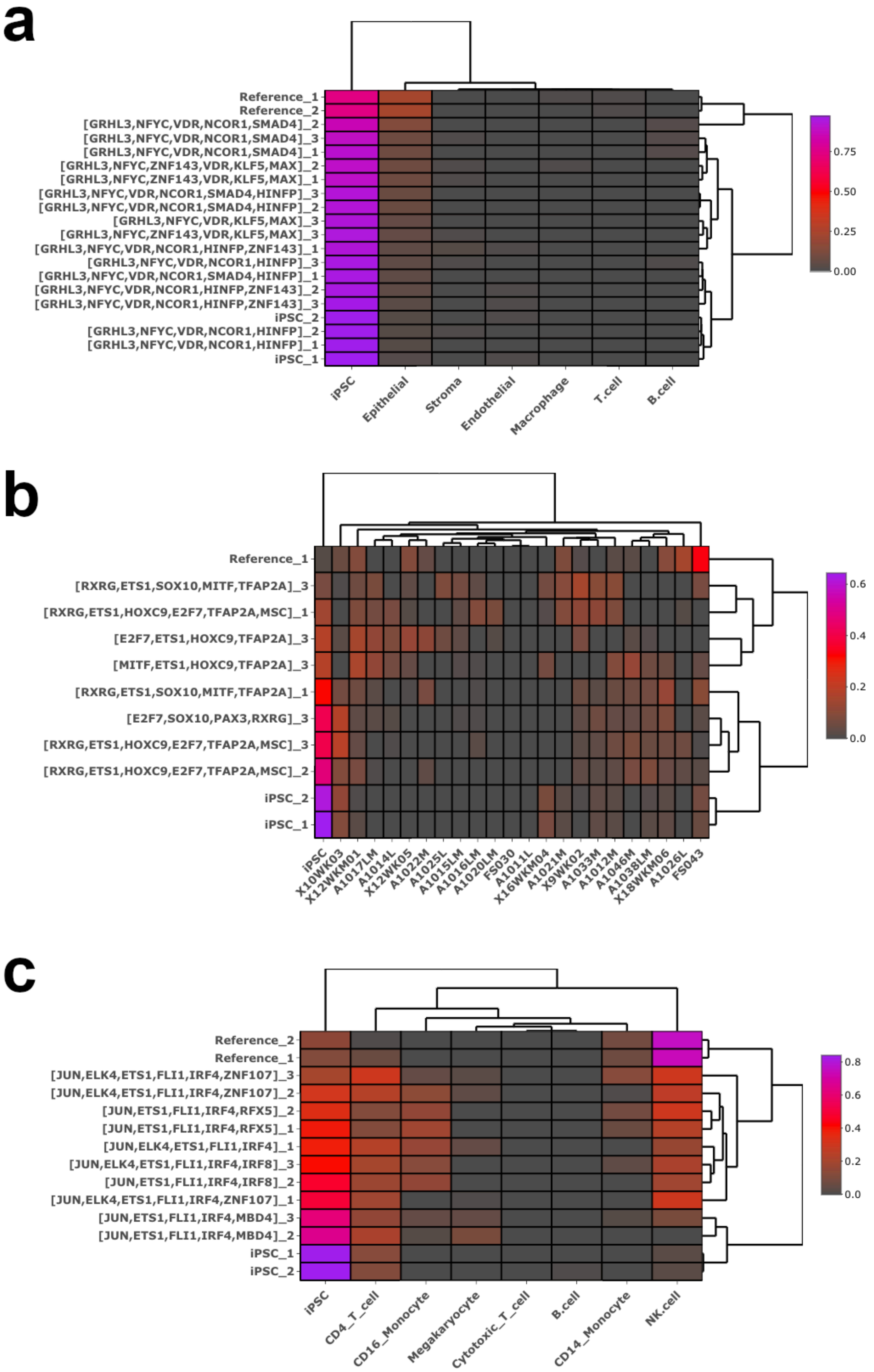

Supplementary Figure 7. Related to Figure 3. Deconvolution of RNA-seq samples using CybersortX. RNA-seq samples of target cell types (starting with Reference), converted cell populations (named after the combination of IFs used for inducing them) and iPS cells (starting with iPSC) have been deconvoluted by CybersortX. The fraction of cells predicted to belong to a cell type is color-coded from low (grey) over medium (red) to high (purple). Reference single-cell RNA-seq samples have been collected for **a.** mammary epithelial cells (breast tumor cells), **b.** melanocytes (neonatal, starting with ‘X’ or ‘FS’, and adult skin samples, starting with ‘A’, enriched in melanocytes) and **c.** NK cells (peripheral blood mononuclear cells). Converted cell samples with less than five million reads have been excluded prior to the analysis.

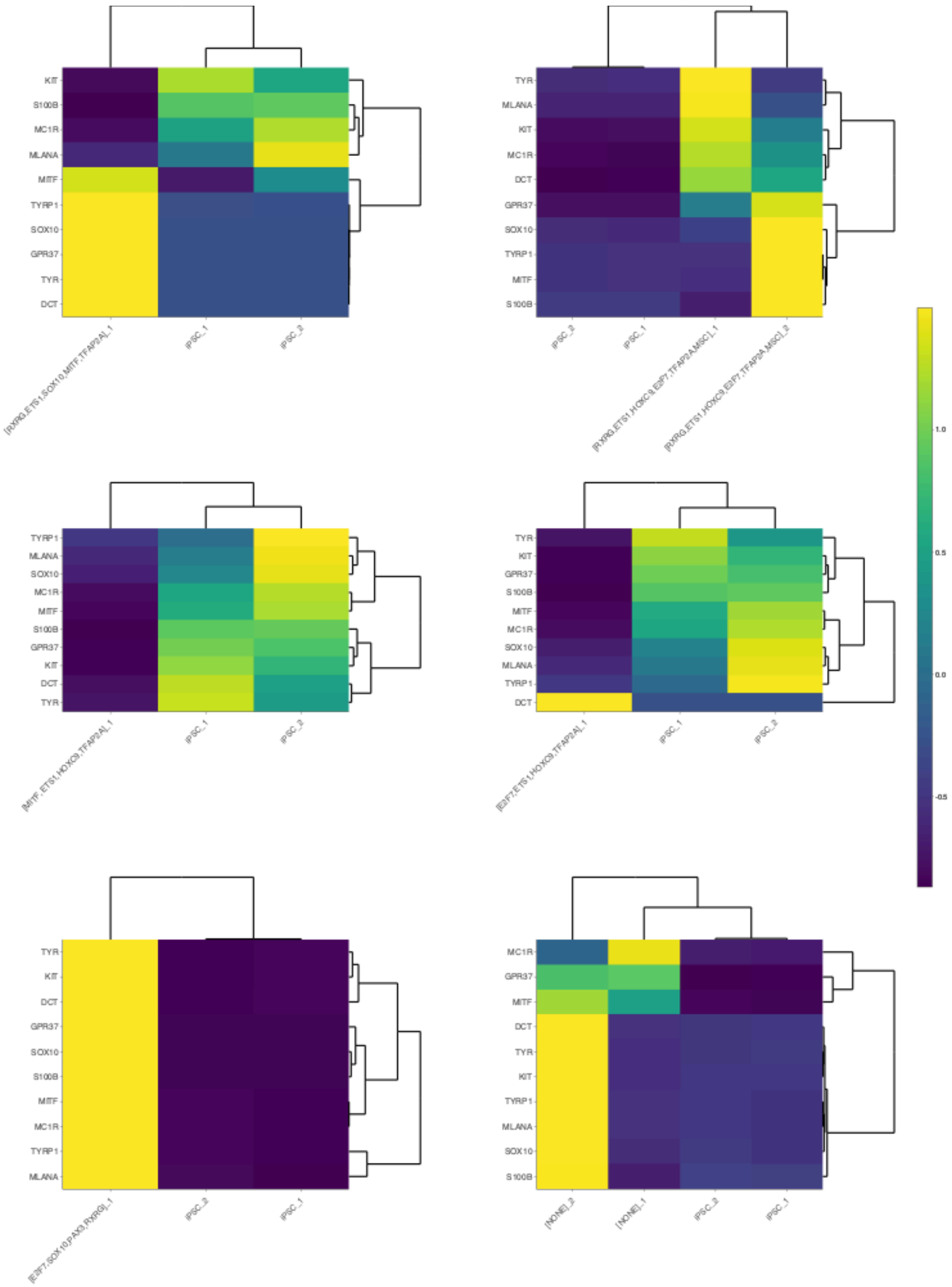

Supplementary Figure 8. Related to Figure 3. Melanocyte marker gene expression. Gene expression from RNA-seq samples of converted cell populations are compared to iPSCs, the initial cell type of the conversion. Heatmaps show scaled expression values of collected marker genes for up- (yellow) and down-regulated (blue) genes. Scaling was performed row-wise. Combinations of IFs are denoted in brackets. Multiple replicates from the same cell population are marked by an underscore followed by a sequential number. Only samples having more than 5 million reads were considered for this assessment.

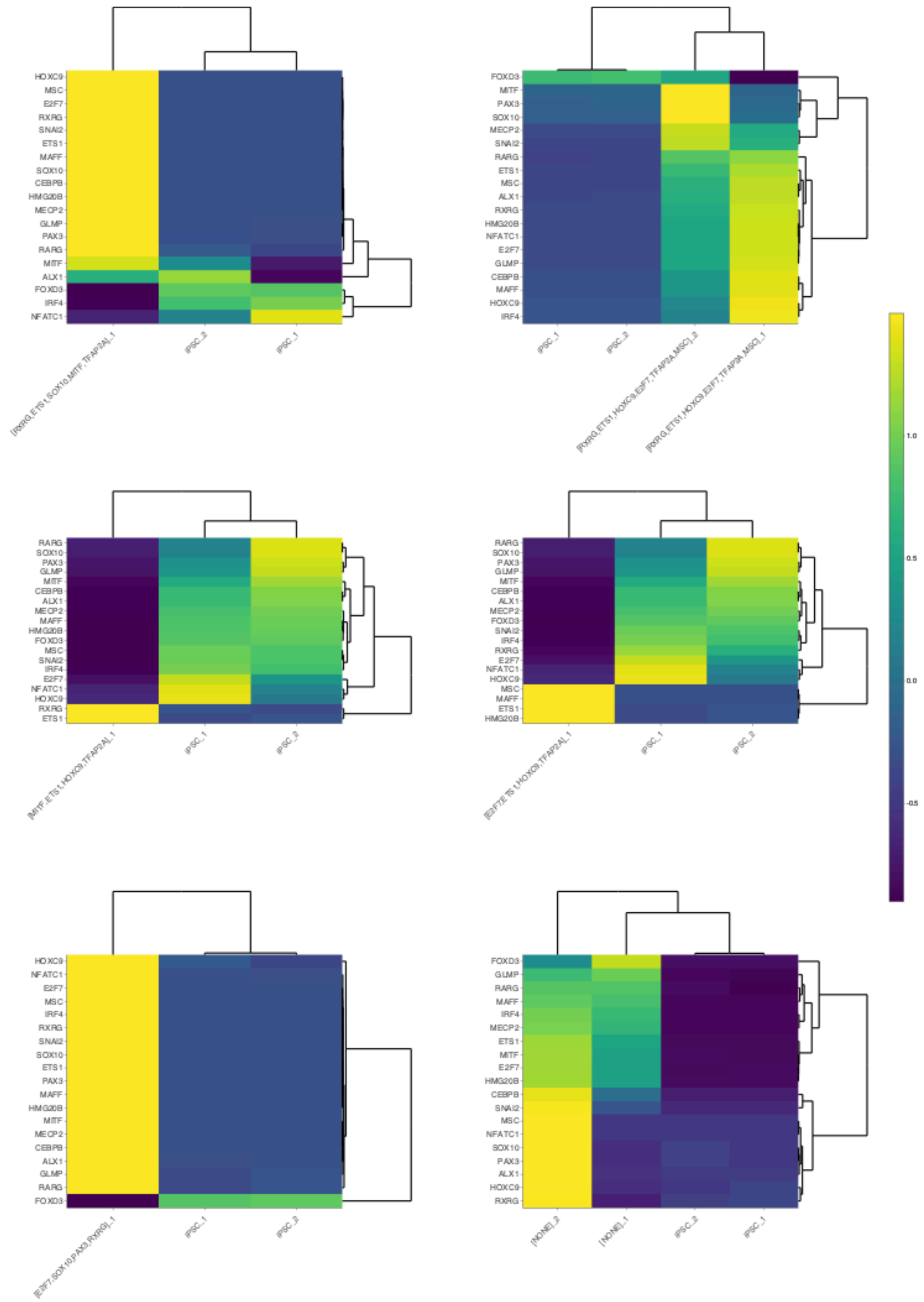

Supplementary Figure 9. Related to Figure 3. Melanocyte network TF gene expression. Gene expression from RNA-seq samples of converted cell populations are compared to iPSCs, the initial cell type of the conversion. Heatmaps show scaled expression values of TFs present in the melanocyte network reconstructed by IRENE for up- (yellow) and down-regulated (blue) genes. Combinations of IFs are denoted in brackets. Scaling was performed row-wise. Multiple replicates from the same cell population are marked by an underscore followed by a sequential number. Only samples having more than 5 million reads were considered for this assessment.

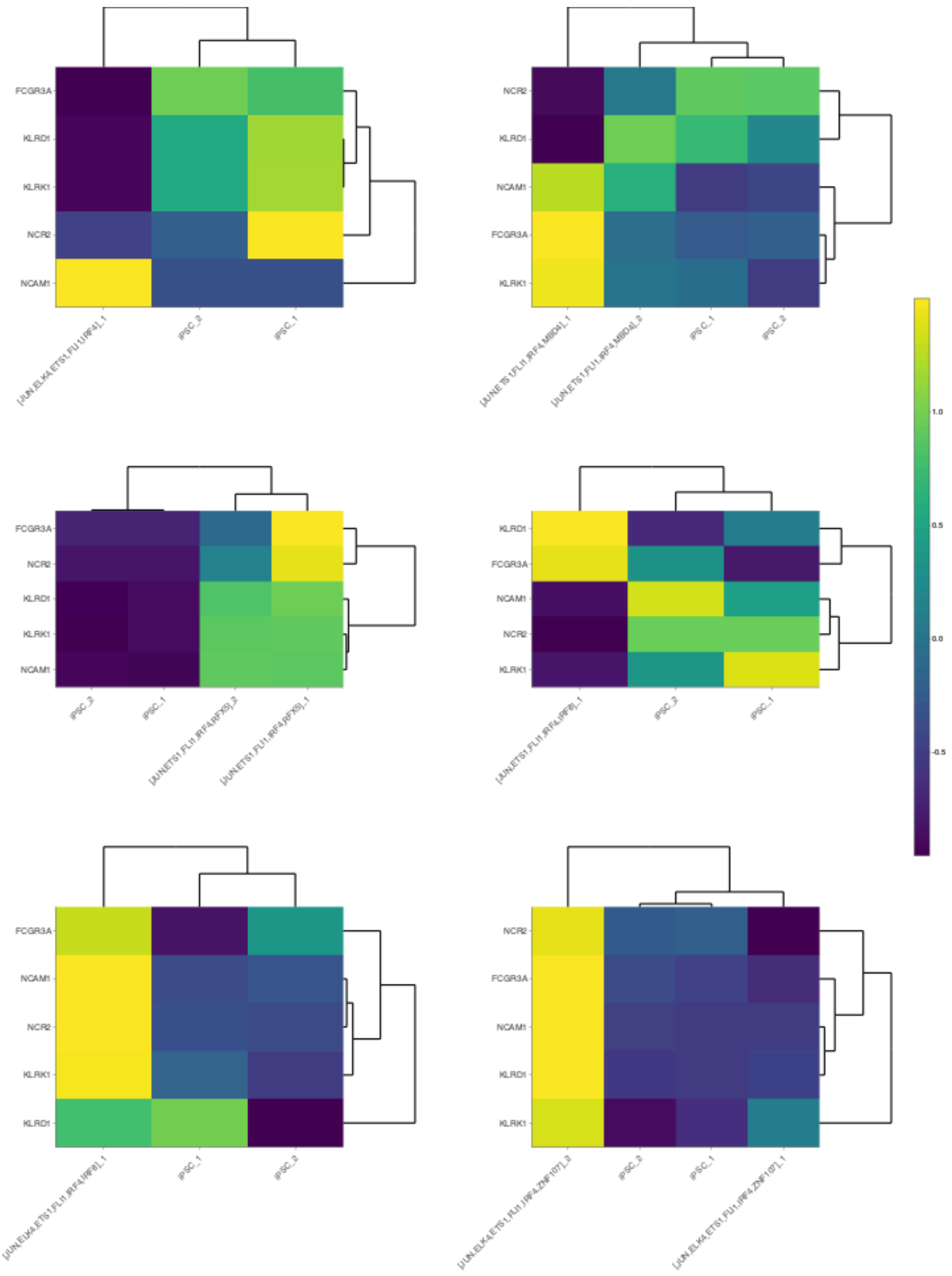

Supplementary Figure 10. Related to Figure 3. NK-cell marker gene expression. Gene expression from RNA-seq samples of converted cell populations are compared to iPSCs, the initial cell type of the conversion. Heatmaps show scaled expression values of collected marker genes for up- (yellow) and down-regulated (blue) genes. Scaling was performed row-wise. Combinations of IFs are denoted in brackets. Multiple replicates from the same cell population are marked by an underscore followed by a sequential number. Only samples having more than 5 million reads were considered for this assessment.

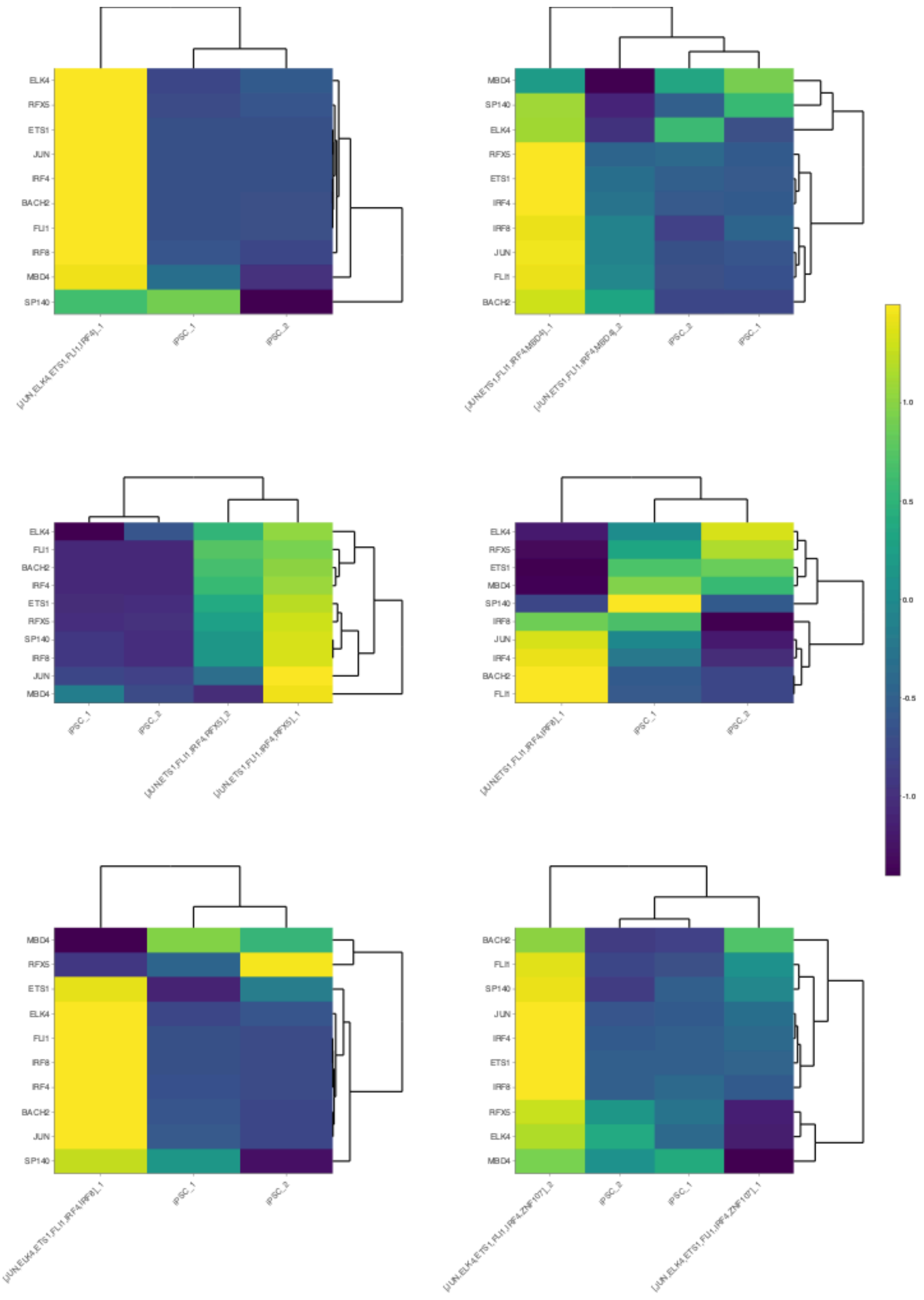

Supplementary Figure 11. Related to Figure 3. NK-cell network TF gene expression. Gene expression from RNA-seq samples of converted cell populations are compared to iPSCs, the initial cell type of the conversion. Heatmaps show scaled expression values of TFs present in the NK-cell network reconstructed by IRENE for up- (yellow) and down-regulated (blue) genes. Combinations of IFs are denoted in brackets. Scaling was performed row-wise. Multiple replicates from the same cell population are marked by an underscore followed by a sequential number. Only samples having more than 5 million reads were considered for this assessment.

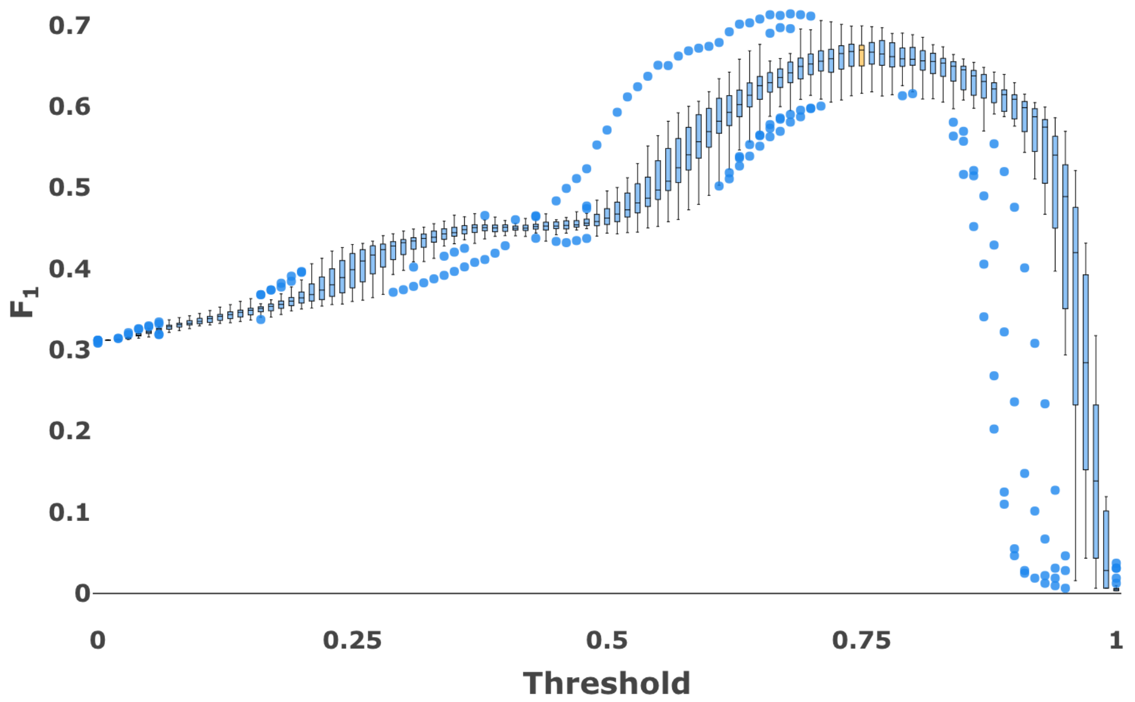

Supplementary Figure 12. Related to Figure 1. Comparison of correlation thresholds for distinguishing ESC from non-ESC samples. The F_1 score for distinguishing random sets of 30 ESC and non-ESC transcriptomic samples using different Pearson correlation thresholds. Each box represents 100 randomly chosen sets of samples. The highest median F_1 score has been obtained for a correlation threshold of 0.75 (yellow). The ends of the box represent the lower and upper quartiles, while the median is marked by a line inside the box. Whiskers extend to 1.5-times the interquartile range. Dots represent outliers.

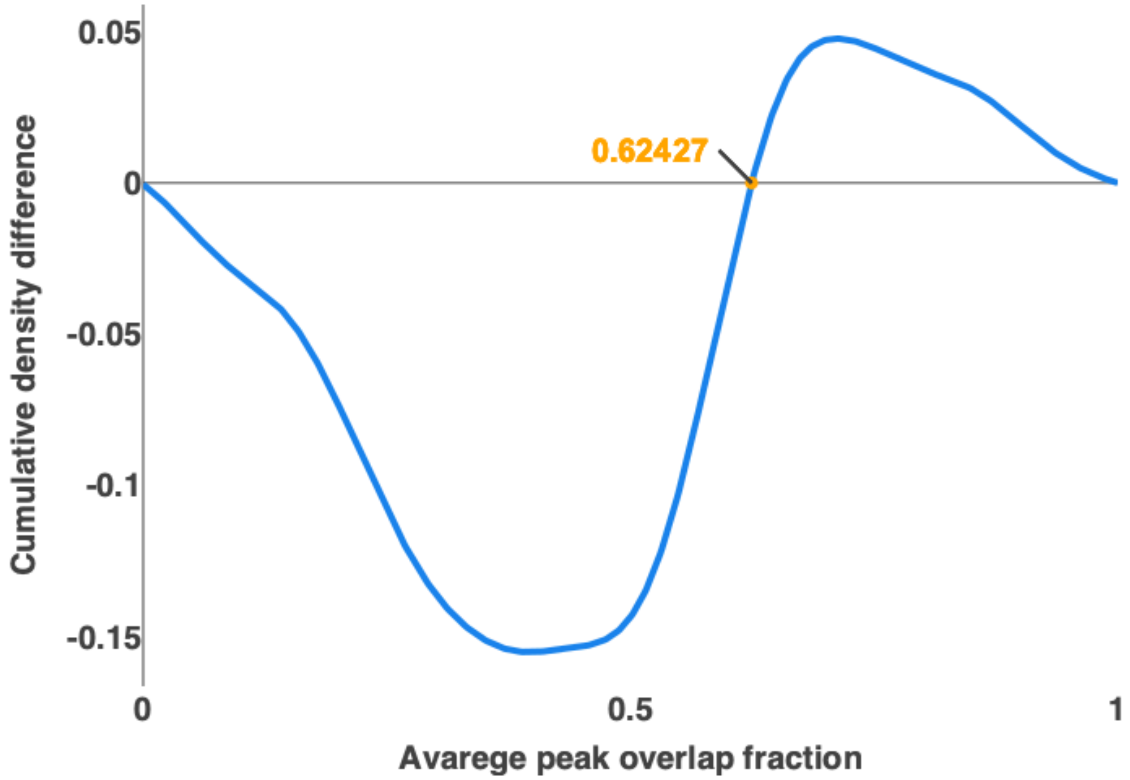

Supplementary Figure 13. Related to Figure 1. Detection threshold for cooperative TFs. The line shows the cumulative probability differences of true positive and true negative protein-protein-interactions (PPIs). Differences are defined over the range of reciprocal TF ChIP-seq peak overlap fractions between two (non-)interacting TFs. Peaks reciprocally overlapping by at least 0.62 (orange dot) are more likely to be truly interacting.

SUPPLEMENTARY REFERENCES

1. Odom, D. T. *et al.* Core transcriptional regulatory circuitry in human hepatocytes. *Mol. Syst. Biol.* **2**, (2006).
2. Boyer, L. A. *et al.* Core Transcriptional Regulatory Circuitry in Human Embryonic Stem Cells. *Cell* **122**, 947–956 (2005).
3. Fournier, M. *et al.* FOXA and master transcription factors recruit Mediator and Cohesin to the core transcriptional regulatory circuitry of cancer cells. *Sci. Rep.* **6**, 34962 (2016).
4. Oki, S. *et al.* ChIP -Atlas: a data-mining suite powered by full integration of public Ch IP -seq data. *EMBO Rep.* **19**, (2018).
5. Zhao, H. *et al.* CrossMap: a versatile tool for coordinate conversion between genome assemblies. *Bioinformatics* **30**, 1006–1007 (2014).
6. Kobayashi, K. & Hiraishi, K. Verification and Optimal Control of Context-Sensitive Probabilistic Boolean Networks Using Model Checking and Polynomial Optimization. *Sci. World J.* **2014**, (2014).
