# Supplementary material for: A computer-guided design tool to increase the efficiency of cellular conversions
Source: Nat Commun. 2021 Mar 12;12:1659. doi: 10.1038/s41467-021-21801-4 (PMC7954801; doi:10.1038/s41467-021-21801-4)
Supplement: Supplementary file 2 — Description of Additional Supplementary Files [file 41467_2021_21801_MOESM2_ESM.pdf]

Description of Additional Supplementary Files

File Name: Supplementary Data 1
Description: **Related to Figure 1. Gene Ontology enrichment of Network TFs.** Each sheet contains all significant gene ontology (GO) terms sorted by their enrichment. Enrichment was only performed on terms classified in the "Biological Process" category. Out of 72 cell types, cell lines and tissue types, seven did not show any enrichment (Sheet "No enrichment")

File Name: Supplementary Data 2
Description: **Related to Figure 1. Gold standard network comparison with IRENE.** Comparison of reconstructed networks using Cistrome and GeneHancer or EnhancerAtlas and GTRD for enhancer-gene associations and transcriptional regulatory interactions, respectively.

File Name: Supplementary Data 3
Description: **Related to Figure 1. Transcriptomics datasets from Recount 2.** List of all 7600 Gene Expression Omnibus accessions of transcriptomics samples from Recount 2 that were used for the identification of identity TFs. No samples from The Cancer Genome Atlas and those containing the terms "cancer", "disease" or "single cell" in their title or description were considered.

File Name: Supplementary Data 4
Description: **Related to Figure 1. Datasets for network reconstruction.** For each processed cell type, accession numbers to the RNA-seq, DNas-seq, H3K27ac ChIP-seq and H3K4me3 ChIP-seq datasets are provided. Replicates datasets are separated by underscores while distinct datasets appear in separate lines having the same cell type, cell line or tissue type identifier.

File Name: Supplementary Data 5
Description: **Related to Table 1. TF ChIP-seq datasets from Gene Expression Omnibus.** List of all TF ChIP-seq experiments considered for reconstructing gene regulatory networks. For each dataset, the Gene Expression Omnibus accession, the profiled cell type and the profiled TF are provided.

File Name: Supplementary Data 6
Description: **Related to Figure 1. Collection of predicted IFs for cellular conversions.** 3365 predicted IF combinations for various initial and target cell types. For each conversion, the top 5 combinations were reported.
