# Supplementary material for: A computer-guided design tool to increase the efficiency of cellular conversions
Source: Nat Commun. 2021 Mar 12;12:1659. doi: 10.1038/s41467-021-21801-4 (PMC7954801; doi:10.1038/s41467-021-21801-4)
Supplement: Supplementary file 9 — Reporting Summary [file 41467_2021_21801_MOESM9_ESM.pdf]

nature research

Corresponding author(s): Antonio del Sol

Last updated by author(s): Feb 3, 2021

Reporting Summary

Nature Research wishes to improve the reproducibility of the work that we publish. This form provides structure for consistency and transparency in reporting. For further information on Nature Research policies, see Authors & Referees and the Editorial Policy Checklist.

Statistics

For all statistical analyses, confirm that the following items are present in the figure legend, table legend, main text, or Methods section.

n/a	Confirmed	
☐	☒	The exact sample size (*n*) for each experimental group/condition, given as a discrete number and unit of measurement
☒	☐	A statement on whether measurements were taken from distinct samples or whether the same sample was measured repeatedly
☐	☒	The statistical test(s) used AND whether they are one- or two-sided<br>*Only common tests should be described solely by name; describe more complex techniques in the Methods section.*
☒	☐	A description of all covariates tested
☐	☒	A description of any assumptions or corrections, such as tests of normality and adjustment for multiple comparisons
☐	☒	A full description of the statistical parameters including central tendency (e.g. means) or other basic estimates (e.g. regression coefficient) AND variation (e.g. standard deviation) or associated estimates of uncertainty (e.g. confidence intervals)
☐	☒	For null hypothesis testing, the test statistic (e.g. *F*, *t*, *r*) with confidence intervals, effect sizes, degrees of freedom and *P* value noted<br>*Give P values as exact values whenever suitable.*
☒	☐	For Bayesian analysis, information on the choice of priors and Markov chain Monte Carlo settings
☒	☐	For hierarchical and complex designs, identification of the appropriate level for tests and full reporting of outcomes
☐	☒	Estimates of effect sizes (e.g. Cohen's *d*, Pearson's *r*), indicating how they were calculated

Our web collection on statistics for biologists contains articles on many of the points above.

Software and code

Policy information about availability of computer code

Data collection	No software was used to collect the data used in this manuscript.
Data analysis	- Transcriptomics datasets were discretized using RefBool (https://github.com/saschajung/RefBool) with Matlab 2018a<br>- Gene Ontology enrichment was performed using the WebgestaltR R-package (version 0.4.2)<br>- Model checking was performed using PRISM v4.4 (https://www.prismmodelchecker.org/)<br>- Transcriptional regulatory networks were reconstructed using IRENE as described in the manuscript (https://github.com/saschajung/IRENE)<br>- IRENE was employed using the Igraph R-package v1.2.2 and Bedtools v2.22.1 (https://github.com/arq5x/bedtools2/releases/tag/v2.22.1)<br>- ChIP-seq datasets used in this study were lifted to genome assembly GRCh38 from hg19 whenever necessary using CrossMap v0.3.4 (http://crossmap.sourceforge.net/)<br>- IRENE was developed and tested under R 3.6.1<br>- Flow cytometry data was analyzed using the flowCore R-package v1.52.1 and related packages [grappolo_0.5.1, flowStats_3.44.0, svglite_1.2.3.2, lattice_0.20-41,gridExtra_2.3, ggcyto_1.14.1, flowWorkspace_3.34.1, ncdfFlow_2.32.0, BH_1.72.0-3, RcppArmadillo_0.9.900.2.0, ggplot2_3.3.2]

For manuscripts utilizing custom algorithms or software that are central to the research but not yet described in published literature, software must be made available to editors/reviewers. We strongly encourage code deposition in a community repository (e.g. GitHub). See the Nature Research guidelines for submitting code & software for further information.

Data

Policy information about availability of data

All manuscripts must include a data availability statement. This statement should provide the following information, where applicable:

- Accession codes, unique identifiers, or web links for publicly available datasets
- A list of figures that have associated raw data
- A description of any restrictions on data availability

The RNA-seq data generated in this study can be found in GEO: GSE165961 [https://www.ncbi.nlm.nih.gov/geo/query/acc.cgi?acc=GSE165961]. Accession numbers of transcriptomics datasets used for identifying identity TFs are provided in Supplementary Data 3. Accession numbers of datasets employed for reconstructing gene regulatory networks can be found in Supplementary Data 4. TF ChIP-seq accession numbers for network reconstruction are provided in Supplementary Data 5. Supplementary Data 6 contains pre-computed combinations of IFs for various cellular conversions. Databases used throughout this study are publicly available: Eukaryotic Promoter Database [https://epd.epfl.ch/human/human_database.php?db=human], AnimalTFDB v3 [http://bioinfo.life.hust.edu.cn/static/AnimalTFDB3/download/Homo_sapiens_TF], GeneHancer v4.7 [https://genecards.weizmann.ac.il/geneloc_prev/genehancer.xlsx], Chip Atlas [https://chip-atlas.org/peak_browser], iRefIndex [https://irefindex.vib.be/] and Recount2 [https://jhubiostatistics.shinyapps.io/recount/]. The datasets used for generating single-cell RNA-seq reference samples for bulk RNA-seq deconvolution are publicly available in GEO: iPSC [https://www.ebi.ac.uk/arrayexpress/files/E-MTAB-6819/E-MTAB-6819.processed.1.zip], breast tumor tissue [https://www.ncbi.nlm.nih.gov/geo/query/acc.cgi?acc=GSE118389], PBMCs [https://www.ncbi.nlm.nih.gov/geo/query/acc.cgi?acc=GSE132044] and melanocytes [https://www.ncbi.nlm.nih.gov/geo/query/acc.cgi?acc=GSE151091].

Field-specific reporting

Please select the one below that is the best fit for your research. If you are not sure, read the appropriate sections before making your selection.

☒ Life sciences ☐ Behavioural & social sciences ☐ Ecological, evolutionary & environmental sciences

For a reference copy of the document with all sections, see nature.com/documents/nr-reporting-summary-flat.pdf

Life sciences study design

All studies must disclose on these points even when the disclosure is negative.

Sample size	All cell lines that were made with transcription factor combinations were determined from lists generated by the software tool. The number of combinations tested (i.e. sample size) was a direct result of outputs from the software tool, which were variable in size.
Data exclusions	No data was excluded.
Replication	All data was gathered for 3 experimental biological replicates.
Randomization	This study did not require randomization - it was limited to engineering cell lines and measuring and observing changes compared to controls.
Blinding	Blinding was not relevant as there were no human participants, only scientific measurements of engineered cells.

Reporting for specific materials, systems and methods

We require information from authors about some types of materials, experimental systems and methods used in many studies. Here, indicate whether each material, system or method listed is relevant to your study. If you are not sure if a list item applies to your research, read the appropriate section before selecting a response.

Materials & experimental systems

n/a	Involved in the study	
☐	☒	Antibodies
☐	☒	Eukaryotic cell lines
☒	☐	Palaeontology
☒	☐	Animals and other organisms
☒	☐	Human research participants
☒	☐	Clinical data

Methods

n/a	Involved in the study	
☒	☐	ChIP-seq
☐	☒	Flow cytometry
☒	☐	MRI-based neuroimaging

Antibodies

Antibodies used

EpCAM-PE Cy 7, Source: Biolegend, Identifier: 369816
Erb2-APC Vio 777, Source: Miltenyi, Identifier: 130-106-756
CD56-APC, Source: Biolegend, Identifier: 318310
CD16-PerCP Cy 5.5, Source: Fisher Scientific, Identifier: 16-0167-82
NKG2D-FITC, Source: Biolegend, Identifier: 320820

NKp44-PE, Source: Fisher Scientific, Identifier: 17-3369-42
NKp46-PE Cy 7, Source: Becton & Dickson, Identifier: 562101
CD26-PerCP Cy 5.5, Source: Biolegend, Identifier: 302716
Mel.2-anti mouse IgG1, Source: abcam, Identifier: ab128759
PE Mouse IgG1, Source: Becton & Dickson, Identifier: 555749

Validation

All antibodies were individually validated by the manufacturer for reactivity to human cells of relevant cell types (NK-cells, melanocytes and epithelial cells). Additional information pertaining to validation can be found on the manufacturer's websites.

Eukaryotic cell lines

Policy information about cell lines

Cell line source(s)

Genetically modified cell lines were all derived from induced pluripotent stem cell line PGP1 (https://www.coriell.org/0/Sections/Search/Sample_Detail.aspx?Ref=GM23338&Product=CC). This cell line was modified to include combination of transcription factor over-expression cassettes as outlined in the methods section of the paper.]. PGP1 cell lines were modified to incorporate transcription factor over-expression cassettes into the genome to create cell lines. The following cell lines were created (ex: "Cell line name [TF1, TF2,...]"): NK 5.1 [JUN,ELK4,ETS1,FLI1,IRF4]; NK 5.2 [JUN,ETS1,FLI1,IRF4,MBD4]; NK 5.3 [JUN,ETS1,FLI1,IRF4,RFX5]; NK 5.4 [JUN,ETS1,FLI1,IRF4,IRF8]; NK 5.5 [JUN,BACH2,ETS1,FLI1,IRF4]; NK 6.1 [JUN,ELK4,ETS1,FLI1,IRF4,IRF8]; NK 6.2 [JUN,ELK4,ETS1,FLI1,IRF4,ZNF107]; NK 6.4 [JUN,ELK4,ETS1,FLI1,IRF4,MBD4]; Mel L [E2F7,SOX10,PAX3,RXRG]; Mel H1 [MITF,ETS1,HOXC9,TFAP2A]; Mel H2 [E2F7,ETS1,HOXC9,TFAP2A]; Mel 5.1 [RXRG,ETS1,SOX10,MITF,TFAP2A]; Mel 6.1 [RXRG,ETS1,HOXC9,E2F7,TFAP2A,MSC]; HMEC 5.1 [GRHL3,NFYC,VDR,KLF5,MAX]; HMEC 5.2 [GRHL3,NFYC,VDR,NCOR1,HINFP]; HMEC 5.3 [GRHL3,NFYC,VDR,SMAD4,HINFP]; HMEC 5.4 [GRHL3,NFYC,VDR,NCOR1,SMAD4]; HMEC 5.5 [GRHL3,HINFP,ZNF143,SMAD2,CREB1]; HMEC 6.1 [GRHL3,NFYC,ZNF143,VDR,KLF5,MAX]; HMEC 6.2 [GRHL3,NFYC,VDR,NCOR1,SMAD4,HINFP]; HMEC 6.3 [GRHL3,NFYC,VDR,NCOR1,HINFP,ZNF143]; HMEC 6.4 [GRHL3,HINFP,ZNF143,SMAD2,CREB1,NCOR1].

Authentication

PGP1 cell line authenticated prior to use in this study. No additional authentication was performed in this study.

Mycoplasma contamination

Cell lines tested negative for mycoplasma contamination.

Commonly misidentified lines
(See ICLAC register)

No misidentified cell lines were used.

Flow Cytometry

Plots

Confirm that:

☒ The axis labels state the marker and fluorochrome used (e.g. CD4-FITC).

☒ The axis scales are clearly visible. Include numbers along axes only for bottom left plot of group (a 'group' is an analysis of identical markers).

☒ All plots are contour plots with outliers or pseudocolor plots.

☒ A numerical value for number of cells or percentage (with statistics) is provided.

Methodology

Sample preparation

All samples were cultured from PGP1 stem cells (https://www.coriell.org/0/Sections/Search/Sample_Detail.aspx?Ref=GM23338&Product=CC). Additional culturing details can be found in the methods section of the paper.

Instrument

Data collected as FCS 3.0 files from a BD LSR Fortessa Cell Analyzer.

Software

R Bioconductor packages were used to analyze the data. Specifically, the 'flowCore' and related packages were used to analyze FCS files.

Cell population abundance

All samples collected at least 10,000 events and were not sorted prior to analysis.

Gating strategy

Gating was performed only at the last step in comparison to negative controls from stained iPS cells that had not been treated with a drug to induce transcription factor expression. In addition, we also had media controls where stem cells were grown in complex medias without transcription factors to use as a comparison of the results to a cell line cultured the same way without transcription factors when relevant.

☒ Tick this box to confirm that a figure exemplifying the gating strategy is provided in the Supplementary Information.
